# Aromatic S_N_^F^-Approach to Fluorinated Phenyl *tert*-Butyl Nitroxides

**DOI:** 10.3390/molecules24244493

**Published:** 2019-12-08

**Authors:** Evgeny Tretyakov, Pavel Fedyushin, Elena Panteleeva, Larisa Gurskaya, Tatyana Rybalova, Artem Bogomyakov, Elena Zaytseva, Maxim Kazantsev, Inna Shundrina, Victor Ovcharenko

**Affiliations:** 1 N. N. Vorozhtsov Institute of Organic Chemistry, 9 Ac. Lavrentiev Avenue, 630090 Novosibirsk, Russia; feduyshin@nioch.nsc.ru (P.F.); pantel@nioch.nsc.ru (E.P.); gurlar82@nioch.nsc.ru (L.G.); rybalova@nioch.nsc.ru (T.R.); elena@nioch.nsc.ru (E.Z.); kazancev@nioch.nsc.ru (M.K.); ishund@nioch.nsc.ru (I.S.); 2 Novosibirsk State University, 2 Pirogova Str., 630090 Novosibirsk, Russia; bus@tomo.nsc.ru; 3 International Tomography Center, 3a Institutskaya Str., 630090 Novosibirsk, Russia

**Keywords:** fluoroarenes, aromatic nucleophilic substitution, *tert*-butylanilines, nitroxides, copper-nitroxide complexes, magneto-structural correlations

## Abstract

The interaction of octafluorotoluene (**1a**), as well as pentafluorobenzonitrile (**1b**) with *tert*-butylamine, followed by the oxidation of thus formed *tert*-butylanilines (**2a**,**b**) with *meta*-chloroperoxybenzoic acid led to functionalized perfluorinated phenyl *tert*-butyl nitroxides [namely, 4-(*N*-*tert*-butyl(oxyl)amino)heptafluorotoluene (**3a**) and 4-(*N*-*tert*-butyl(oxyl)amino)tetrafluorobenzonitrile (**3b**)] with nearly quantitative total yields. The molecular and crystal structures of nitroxide **3a** were proved by single crystal X-ray diffraction analysis. The radical nature of both nitroxides was confirmed by ESR data. The interaction of Cu(hfac)_2_ with the obtained nitroxides **3a**,**b** gave corresponding *trans*-bis(1,1,1,5,5,5-hexafluoropentane-2,4-dionato-*κ*^2^*O*,*O*′)bis{4-(*N*-*tert*-butyl(oxyl)amino)perfluoroarene-*κO*}copper (II) complexes ([Cu(hfac)_2_(**3a**)_2_] and [Cu(hfac)_2_(**3b**)_2_]). X-ray crystal structure analysis showed square bipyramid coordination of a centrally symmetric Cu polyhedron with the axial positions occupied by oxygen atoms of the nitroxide groups. Magnetic measurements revealed intramolecular ferromagnetic exchange interactions between unpaired electrons of Cu(II) ions and paramagnetic ligands, with exchange interaction parameters *J_Cu–R_* reaching 53 cm^−1^.

## 1. Introduction

The use of organofluorine compounds has had a huge impact on the main areas of modern chemistry, such as the development of new functional materials and pharmaceuticals with unique properties [1,2,3,4]. The ever-increasing importance of fluorine organic compounds brings the development of new directed and high-performance synthetic methods to the forefront [5]. The chemistry of stable organic radicals is among the scientific fields where the need for the creation of new methods for the synthesis of fluorinated compounds is ripe. The number of studies here is extremely limited, although interest in fluorinated organic radicals has been consistently strong [6,7,8,9]. In particular, this interest is due to the unusual magnetic and functional properties that fluorinated paramagnets often manifest [10,11,12,13]. To devise new approaches to their synthesis, we applied nucleophilic substitution reactions of a fluorine atom in a series of polyfluorinated aromatic compounds. This idea has been especially fruitful in nitroxide chemistry. For example, the 4,4,5,5-tetramethyl-4,5-dihydro-1*H*-imidazole-3-oxide-1-oxyl lithium derivative has been shown to react with polyfluoroaromatic compounds and give rise to new fluorinated nitronyl nitroxides [14,15,16]. It is noteworthy that according to quantum chemical calculations, the reaction follows a concerted pathway without formation of an intermediate [14]. The reason is that at an early stage, the lithium ion facilitates fluoride elimination when the C–C bond between nitroxide and aromatic carbon atoms is formed only partially.

*tert*-Butyl aryl nitroxides and polynitroxides represent an important class of paramagnets that have been actively used as ligands for the construction of heterospin systems. The design of polyradicals with >N–O groups at the *meta* position of the benzene ring, which favors intraligand ferromagnetic coupling, and their complexation with M(hfac)_2_ have allowed Iwamura et al. to obtain a series of molecular magnets capable of cooperative magnetic ordering at 3.4–46 K [17,18,19,20]. In addition, nitroxides containing aryl fragment substituents able to form intermolecular H-bonds are attractive as components of the controlled assembly of high-dimension systems [21,22,23,24,25,26,27].

**Scheme 1:**
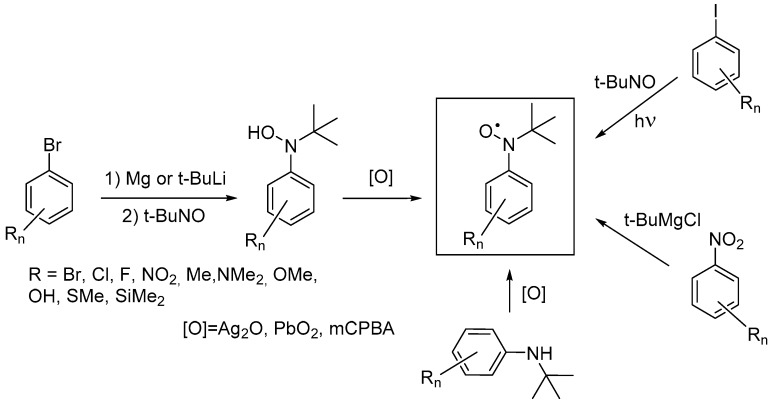
Synthetic approaches to phenyl *tert*-butyl nitroxides.

A typical method for the preparation of *tert*-butyl aryl nitroxides involves oxidation of the corresponding *tert*-butyl anilines or phenylhydroxylamines. As a rule, the general synthetic approach starts with a reaction of an appropriate aromatic organometallic compound with *tert*-nitrosobutane, thus providing aryl *tert*-butylhydroxylamine, followed by its oxidation to the target radical product [28,29]. The methods for preparation of radicals on the basis of UV irradiation of the corresponding iodo arenes in the presence of *tert*-nitrosobutane or the interaction of nitro arenes with *tert*-butyl magnesium chloride (Scheme 1) are also worthy of mention but of no practical value [30,31].

In this study, we have successfully applied the early proposed concept based on nucleophilic substitution of a fluorine atom [14] to the synthesis of fluorinated *N*-*tert*-butyl aryl nitroxides. This approach was found to generate highly stable nitroxide radicals in almost quantitative yields. An interaction of bis(hexafluoroacetylacetonato)copper (II) [abbreviated as Cu(hfac)_2_] with the synthesized nitroxides led to formation of complexes of composition 1:2 containing heterospin clusters (>N–O)_2_Cu.

## 2. Results and Discussion

### 2.1. Synthesis of Fluorinated Nitoxodes ***3a,b***

**Scheme 2:**
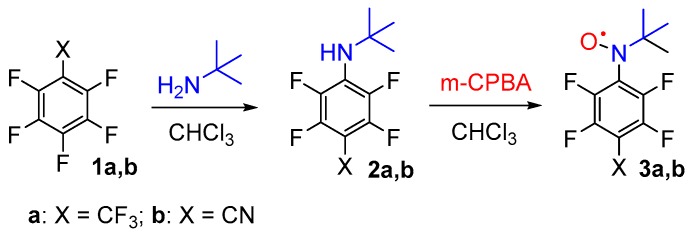
Synthesis of fluorinated nitroxides **3a**,**b**.

As an approach to phenyl *tert*-butyl nitroxides, we linked together two transformations: at the first stage, the nucleophilic substitution of a fluorine atom in an activated arene under the action of *tert*-butylamine, and at the second stage, oxidation of the obtained amine into the target paramagnetic compound. As to the first transformation, it is known that the interaction of perfluorotoluene **1a** with methyl-, *n*-butyl-, *tert*-butylamine or perfluorobenzonitrile **1b** with methylamine in isopropanol at 20–70 °C for 20–90 h leads to selective *para*-fluorine substitution with the formation of the corresponding *N*-alkyl anilines in 70–98% yields [32]. We carried out the reaction of **1a**,**b** with *tert*-butylamine in chloroform at room temperature for 72 and 1.5 h, respectively, and isolated 4-(*tert*-butylamino)heptafluorotoluene **2a** or 4-(*tert*-butylamino)tetrafluorobenzonitrile **2b** in almost quantitative yields. The oxidation of anilines **2a**,**b** with *meta*-chloroperoxybenzoic acid (*m*-CPBA) was performed at room temperature and provided target nitroxides **3a**,**b** as viscous red liquids in yields >90% (Scheme 2).

Newly obtained fluorinated nitroxides **3a**,**b** were comprehensively studied both in solution and in a condensed state.

### 2.2. ESR Measurement of Radicals ***3a,b***

**Figure 1:**
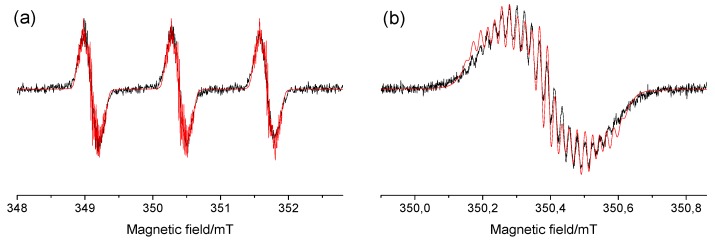
Experimental (black curve) and simulated (red curve) ESR spectra of **3a**: (**a**) the whole spectrum, and (**b**) only the central component.

The ESR spectra for diluted (~10^−4^ M) and oxygen-free chloroform solutions of radicals **3a**,**b** showed triplet patterns at g = 2.0057(2) owing to the hfs of an unpaired electron at the nitrogen nucleus (A_N_ = 1.33 mT for **3a** and 1.31 mT for **3b**; Figure 1 and Figure S18). In the case of nitroxide **3a**, a high-resolution ESR spectrum was recorded to obtain more complex splitting of each line of its triplet. The spectrum was well reproduced, taking into account 9 hfs constants on the protons of tert-butyl group (A_H_ = 0.02 mT) and two pairs of hfs constants on the distant fluorine atoms (A_Fortho_ = 0.12 mT; A_Fmeta_ = 0.06 mT).

### 2.3. Electrocemical Measurements

Electrochemical properties of the studied compounds were evaluated by cyclic voltammetry measurements in a CH_2_Cl_2_ solution (Figure S21). Both radicals **3a** and **3b** demonstrated irreversible oxidation waves with E_1/2_ ≈ 1 V, which were assigned to the oxidation of the nitroxide radicals to the corresponding oxoammonium cations. For comparison, 4-(*N*-*tert*-butyl-*N*-oxylamino)benzene (**4a**) and 4-(*N*-tert-butyl-*N*-oxylamino)benzotrifluoride (**4b**) displayed the reversible redox potentials at 0.38 and 0.53 V, respectively (Table 1). The redox reversibility of **4a** and **4b** could be ascribed to the higher stability of the corresponding oxoammonium cations generated at considerably less oxidative potentials [33].

On the cathodic side, both **3a** and **3b** exhibited an irreversible redox at −1.44 and −1.14 V, respectively. The non-substituted at the phenyl ring nitroxide **4a** also reduced irreversibly, but at a considerably higher potential of −1.63 V, which corresponds to the relative change in the SOMO energy level (Table 1). It is interesting that the trifluoromethylphenyl nitroxide **4b** exhibited redox at −1.37 V in a clear reversible manner that results from the stability of the corresponding aminoxy anion [33]. Therefore, the substitution of phenyl ring influences not only on the redox potentials, but also on the stability of the corresponding anionic forms (oxoammonium cations and aminoxy anions).

### 2.4. Crystal Structure of Radical ***3a***

**Figure 2:**
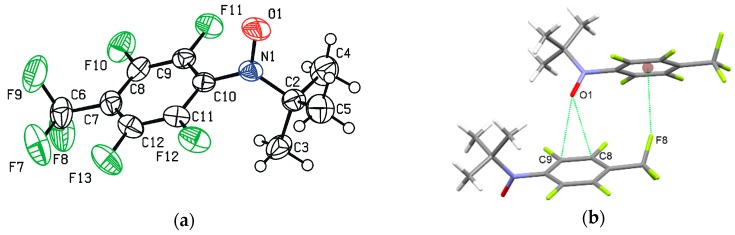
Molecular structure (**a**) and a fragment of the crystal structure (**b**) of nitroxide **3a** (the thermal ellipsoids are drawn at the 50% probability level).

Even though both freshly prepared radicals **3a**,**b** were red oils, repeated efforts were made to obtain them in a crystalline form. Finally, by crystallization from a cold hexane solution, we managed to isolate nitroxide **3a** as high-quality crystals and determined its molecular and crystalline structure by X-ray diffraction (XRD) analysis. The radical **3a** crystallizes in the orthorhombic Pbca space group, and bond lengths of the *tert*-butyl-nitroxide moiety are completely compatible with those of previously described radicals of this family (see Table 2 for crystallographic data). The nitroxide group in radical **3a** is twisted by a large angle (~68°) relative to the aromatic ring (Figure 2), obviously owing to the mutual effects of steric repulsion between the *tert*-butyl group and phenylene *ortho*-fluorines and of the electrostatic repulsion of dipoles C–F and N–O. In this regard, it should be noted that a similar dihedral angle in nonfluorinated *tert*-butyl phenyl nitroxides is twofold smaller and manifests an experimental value of 23–32°. This finding is consistent with the calculated minimum of the heat of formation (MO, B3LYP/6-31G) for the model radical (*N*-*tert*-butyl(oxyl)amino)benzene at a 34° angle [34].

Analysis of the crystal packing of nitroxide **3a** revealed C–F_CF3_…π interactions, with F…C_g_ and D_pln_ distances equal to 3.069(2) and 3.049 Å, respectively, and O_NO_…C short contacts 3.101(3) and 3.130(3) Å (Figure 2), that formally bind molecules into chains along the *b* axis. The chains in turn are packed into layers parallel to plane (*a*, *b*) with contacts F…F equal to 2.650(2) Å, that is, shorter than the sum of van der Waals radii of two fluorine atoms (2.92 Å) [35].

### 2.5. Synthesis and Crystal Structure of Complexes [Cu(hfac)_2_(***3a***)_2_], [Cu(hfac)_2_(***3b***)_2_] and [Cu(hfac)_2_(***2b***)_2_]

**Scheme 3:**
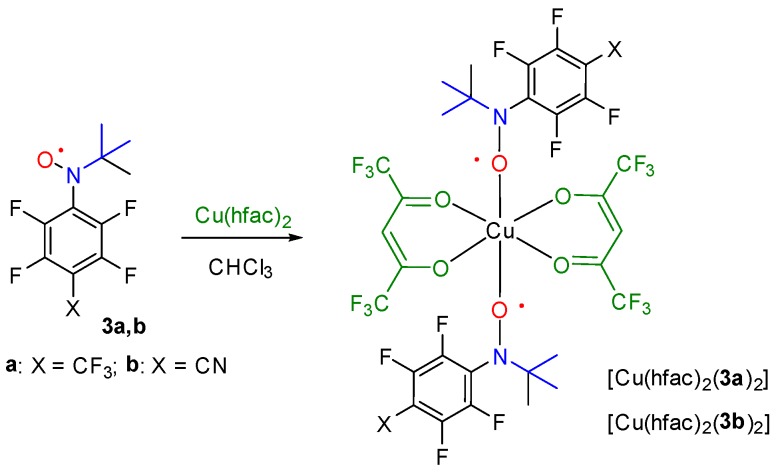
Complexation of Cu(hfac)_2_ with nitroxides **3a**,**b**.

Radicals **3a,b** underwent complexation with half equivalents of Cu(hfac)_2_ in CHCl_3_ to form 1:2 complexes [Cu(hfac)_2_(**3a**)_2_] and [Cu(hfac)_2_(**3b**)_2_] (Scheme 3), which were recrystallized from *n*-hexane to obtain crystals suitable for XRD analysis.

**Figure 3:**
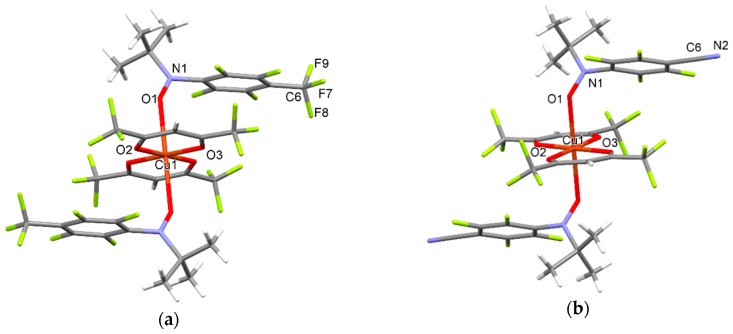
XRD structures of [Cu(hfac)_2_(**3a**)_2_], one of two independent molecules (**a**), and [Cu(hfac)_2_(**3b**)_2_] with omitted minor positions of CF_3_ and *tert*-butyl groups (**b**).

Figure 3 illustrates the molecular structures of [Cu(hfac)_2_(**3a**)_2_] and [Cu(hfac)_2_(**3b**)_2_] (see Table 2 for crystallographic data). The asymmetric part of [Cu(hfac)_2_(**3a**)_2_] includes two halves of independent units located on the centers of symmetry. In the [Cu(hfac)_2_(**3b**)_2_] complex, CF_3_ groups and *tert*-butyl groups are disordered. In the distorted octahedron around the copper ion, two O_NO_ atoms occupy axial positions (*d*_Cu–O_ = 2.398(4), 2.411(5) Å in [Cu(hfac)_2_(**3a**)_2_], and 2.452(4) Å in [Cu(hfac)_2_(**3b**)_2_]). Equatorial Cu–O_hfac_ distances in both complexes do not exceed 1.946(3) Å (Table 3).

Polyfluorinated compounds, including complexes of transition metal ions, are known to have high volatility. Our experiments showed that at reduced pressure (~1 Torr) and a temperature of ~95 °C, at which according to thermal analysis data (Figures S19 and S20) complexes [Cu(hfac)_2_(**3a**)_2_] and [Cu(hfac)_2_(**3b**)_2_] are stable, they almost quantitatively collected on a sublimator finger with the formation of crystalline phases. The infrared (IR) spectra of these phases are fully identical to those of the initial complexes [Cu(hfac)_2_(**3a**)_2_] and [Cu(hfac)_2_(**3b**)_2_]. Moreover, according to the results of the XRD analysis, the structure of the sublimed complexes coincides with that of the starting compounds [Cu(hfac)_2_(**3a**)_2_] and [Cu(hfac)_2_(**3b**)_2_]. This finding indicates that the formation constants of these complexes are quite high, and these compounds are transferred to the gas phase in the form of molecules of coordination compounds with subsequent precipitation.

**Figure 4:**
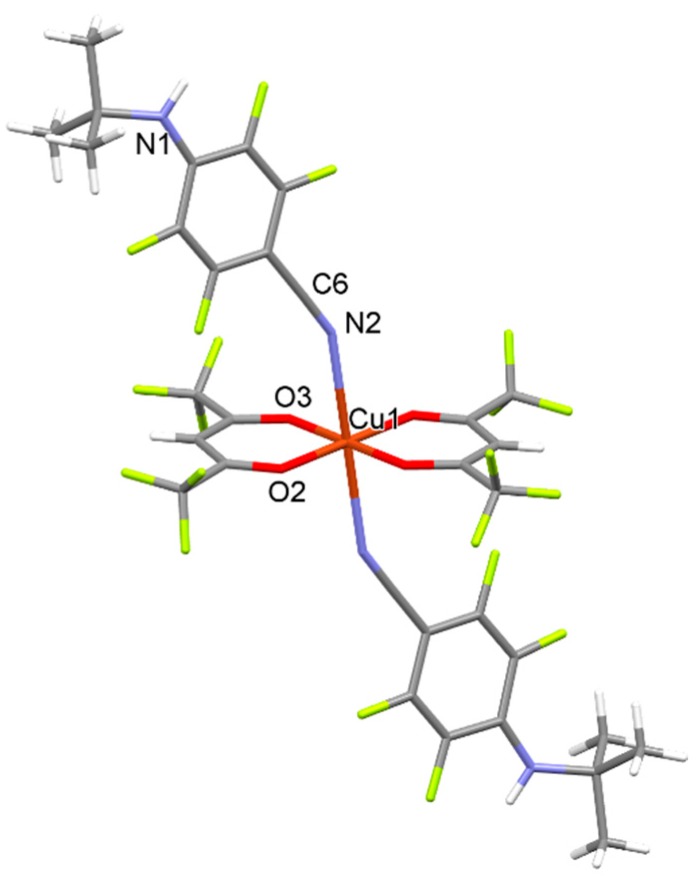
XRD structure of [Cu(hfac)_2_(**2b**)_2_].

Of note, the interaction of Cu(hfac)_2_ with radical **3b** leads to coordination of the nitroxide group, not the nitrile one. Driven by curiosity, we carried out the reaction of acceptor matrix Cu(hfac)_2_ with amine **2b** (Figure S22). In this case, the nitrile group underwent coordination with the formation of a centrosymmetrical mononuclear complex in which two N_CN_ atoms occupy axial positions [*d*_Cu–N_ = 2.534(3) Å] (Figure 4). This means that the nitrile group in **3b** may be also involved in the coordination, as may the nitroxide group, thus affording complexes of chain-polymeric structure.

### 2.6. Magnetic Measurements of Complexes [Cu(hfac)_2_(***3a***)_2_] and [Cu(hfac)_2_(***3b***)_2_]

**Figure 5:**
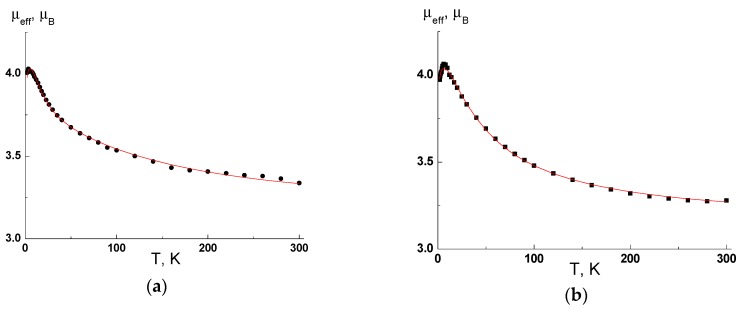
The µ_eff_(*T*) dependences for complexes [Cu(hfac)_2_(**3a**)_2_] (**a**) and [Cu(hfac)_2_(**3b**)_2_] (**b**). Solid lines are theoretical curves.

Temperature dependences of the effective magnetic moment (µ_eff_) for complexes [Cu(hfac)_2_(**3a**)_2_] and [Cu(hfac)_2_(**3b**)_2_] are presented in Figure 5. At 300 K, the µ_eff_ values are 3.28 and 3.34 µ_B_ for [Cu(hfac)_2_(**3a**)_2_] and [Cu(hfac)_2_(**3b**)_2_], respectively, and are in agreement with a theoretical spin-only value of 3 µ_B_ for three paramagnetic centers: one Cu(II) ion and two nitroxides. The increase in µ_eff_ with lowering temperature indicates domination of ferromagnetic exchange interactions between spins of paramagnetic centers. Analysis of experimental µ_eff_(*T*) dependences using a model of a three-spin exchange cluster [spin Hamiltonian *H = −2J_Cu–R_ × (S_R1_S_Cu_ + S_Cu_S_R2_) − 2J_R–R_ × S_R1_S_R2_*] allowed us to estimate exchange interaction parameters. The best-fit values of g_Cu_ and exchange interaction parameters *J_Cu–R_* and *J_R–R_* are 2.26, 53.1 cm^−1^, and −17 cm^−1^ for [Cu(hfac)_2_(**3a**)_2_] and 2.27, 27.0 cm^−1^, and −0.4 cm^−1^ for [Cu(hfac)_2_(**3b**)_2_]. The g-factors for nitroxides were fixed at g_R_ = 2 to avoid overparametrization.

The observed ferromagnetic exchange interactions between the unpaired electrons in [Cu(hfac)_2_(**3a**)_2_] and [Cu(hfac)_2_(**3b**)_2_] are consistent with the XRD data on the axial coordination of the nitroxide groups with the Cu^2+^ ion. According to the results of experimental [36,37,38,39] and theoretical studies [40,41], such coordination provides the orthogonal arrangement of the spins in the exchange cluster with *J_Cu–R_* values, which in some cases exceed 50 cm^−1^. With regard to complexes [Cu(hfac)_2_(**3a**)_2_] and [Cu(hfac)_2_(**3b**)_2_], such a strong ferromagnetic coupling leads to that the half-wave potentials for electrochemical oxidation and reduction are almost the same as those for the free radicals (Table 1).

## 3. Conclusions

Using octafluorotoluene **1a** and pentafluorobenzonitrile **1b** as examples, we demonstrated a new synthetic approach for obtaining functionalized fluorinated phenyl *tert*-butyl nitroxides by sequential substitution of a fluorine atom in polyfluorinated arenes with *tert*-butylamine and oxidation of resultant *tert*-butylaniline with *m*-CPBA. This convenient approach can be useful in the design of nitroxide structures as precursors of new complexes with desired magnetic characteristics. For instance, the interaction of Cu(hfac)_2_ with newly synthesized nitroxides **3a** and **3b** yielded isomorphic three-spin complexes [Cu(hfac)_2_(**3a**)_2_] and [Cu(hfac)_2_(**3b**)_2_]. They are centrosymmetrical square bipyramids with two axial positions occupied by oxygen atoms of the nitroxide groups; this arrangement gives rise to ferromagnetic intramolecular exchange interactions between Cu(II) and radical spins. In complexes with axial coordination of the nitroxyl group observed in compounds [Cu(hfac)_2_(**3a**)_2_] and [Cu(hfac)_2_(**3b**)_2_], thermo- or photo-induced spin transitions may take place [42,43,44,45,46,47]. To obtain such complexes, it is necessary to modify the structure of *tert*-butyl nitroxides of type **3** in an appropriate way. We believe that this may be achieved by a decrease in the number of fluorine atoms on the aromatic ring of paramagnetic ligands **3**.

## 4. Materials and Methods

### 4.1. Reagents and General Methods

Bis(hexafluoroacetylacetonato)copper-(II) [abbreviated as Cu(hfac)_2_] was prepared and purified by previously described procedures [48]. Other chemicals were of the highest purity commercially available and were used as received. The progress of reactions was monitored by thin-layer chromatography (TLC) on Silica gel 60 F_254_ aluminum sheets with hexane or CHCl_3_ as the eluent. Column chromatography was carried out on silica gel (0.063–0.200 mm). NMR spectra were recorded for **2a**,**b** solutions in CDCl_3_ on Bruker Avance-300 (300.13 MHz for ^1^H, 282.25 MHz for ^19^F) and Avance-400 (400.13 MHz for ^1^H, 100.62 MHz for ^13^C) spectrometers; chemical shifts (δ) of ^1^H and ^13^C{1H} are given in ppm, with the solvent signals serving as the internal standard (δH = 7.24 ppm, δC = 76.9 ppm); the internal standard for ^19^F spectra was C_6_F_6_ (δ = −162.9 ppm). Fourier transform infrared (FT-IR) spectra were acquired in KBr pellets on a Bruker Vector-22 spectrometer. UV-vis spectra were registered on an HP Agilent 8453 spectrophotometer (in 10^−5^–10^−4^ M solutions in EtOH). Masses of molecular ions were determined by high-resolution mass spectrometry (HRMS) by means of a DFS Thermo Scientific instrument (EI, 70 eV). Melting points were recorded on a Melter-Toledo FP81 Thermosystem apparatus. Elemental analyses were performed using a Euro EA 3000 elemental analyzer.

### 4.2. Synthesis of 4-(tert-butylamino)arenes ***2a,b*** (General Procedure)

A mixture of *tert*-butyl amine (5.0 mmol, 365 mg) and perfluoroarene **1a** or **1b** (1.0 mmol) in CHCl_3_ (5 mL) was stirred at room temperature until the starting substrate was completely consumed: for 72 and 1.5 h for **1a** and **1b**, respectively. Flash chromatography (SiO_2_, column 3 × 4 cm, CHCl_3_ as an eluent) followed by solvent removal under reduced pressure yielded colorless crystals of pure product **2a** or **2b**.

4-(*tert*-Butylamino)heptafluorotoluene (**2a**) [32]: Colorless crystals; yield 99% (286 mg). ^1^H NMR (300.13 MHz, CDCl_3_): δ 4.07 (s, 1H), 1.37 (s, 9H). ^13^C NMR (125.75 MHz, CDCl_3_): δ 144.94 (dm, *J* = 254.7 Hz, 2C) 137.86 (dm, *J* = 239.3 Hz, 2C), 130.22 (tt, *J_1_* = 12.5 Hz, *J_2_* = 3.2 Hz, 1C) 121.52 (q, *J* = 272.4 Hz, 1C), 97.04–98.08 (m, 1C), 53.76 (s, 1C), 30.46 (t, *J* = 3.7 Hz, 3C). ^19^F NMR (282.37 MHz, CDCl_3_): δ 109.58 (t, *J* = 20.6 Hz, 3F), 21.09–21.48 (m, 2F), 10.67 (d, *J* = 15.9 Hz, 2F). IR (KBr, cm^−1^) 3433, 2978, 2945, 2918, 2879, 1659, 1516, 1470, 1433, 1400, 1371, 1335, 1240, 1203, 1180, 1138, 1043, 993, 930, 872, 804, 714, 422. HRMS calcd for C_11_H_10_F_7_N: 289.0696. Found 289.0698.

4-(*tert*-Butylamino)tetrafluorobenzonitrile (**2b**): Colorless crystals; yield 99% (244 mg). ^1^H NMR (400.13 MHz, CDCl_3_): δ 4.46 (s, 1H), 1.40 (s, 9H). ^13^C NMR (100.62 MHz, CDCl_3_): δ 147.98 (dm, *J* = 256.1 Hz, 2C), 136.13 (dm, *J* = 240.0 Hz, 2C), 132.22 (tt, *J_1_* = 11.9 Hz, *J_2_* = 3.6 Hz, 2C), 108.76 (t, *J* = 3.7 Hz, 2C), 79.76 (tt, *J_1_* = 18.0 Hz, *J_2_* = 1.8 Hz, 1C), 53.85 (s, 1C) 30.60 (s, 1C). ^19^F NMR (282.37 MHz, CDCl_3_): δ 26.34 (m, 2F), 7.68 (d, *J* = 17.4 Hz, 2F). IR (KBr, cm^−1^) 3427, 3020, 2978, 2943, 2881, 2617, 2420, 2233, 1655, 1525, 1510, 1477, 1435, 1400, 1373, 1313, 1302, 1336, 1201, 1176, 1126, 1047, 993, 978, 889, 806, 721, 661, 634, 523, 494. HRMS calcd for C_11_H_10_F_4_N_2_: 246.0775. Found 246.0771. The molecular and crystal structures of **2b** were refined from single-crystal XRD data (see SI).

### 4.3. Synthesis of 4-(N-tert-butyl(oxyl)amino)arenes ***3a,b*** (General Procedure)

A solution of 4-(*tert*-butylamino)perfluoroarene **2a**,**b** (1 mmol) and *m*-CPBA (1.2 mmol, 208 mg) in CHCl_3_ (10 mL) was stirred at room temperature for 48 h. Column chromatography (SiO_2_, column 3 × 20 cm, CHCl_3_ as an eluent) afforded a red fraction of radical **3a**,**b**. The solvent was removed under reduced pressure at room temperature to obtain radicals **3a**,**b**.

4-(*N*-*tert*-butyl(oxyl)amino)heptafluorotoluene (**3a**): Red crystals; yield 96% (292 mg). IR (KBr, cm^−1^) 2987, 2945, 1790, 1766, 1655, 1606, 1504, 1469, 1417, 1358, 1336, 1257, 1232, 1190, 1153, 1197, 991, 904, 831, 791, 715, 588. UV-Vis (EtOH) λ_max_/nm (lg ε): 383 (2.49), 301 (3.30), 271 (3.51), 221 (3.73), 203 (3.93). HRMS calcd for C_11_H_9_O_1_N_1_F_7_: 304.0567. Found 304.0564. Crystals of **3a** were grown from hexane at −15 °C. The molecular and crystal structures of **3a** were refined from single-crystal XRD data (see main text).

4-(*N*-*tert*-butyl(oxyl)amino)tetrafluorobenzonitrile (**3b**): Red oil; yield 95% (248 mg). IR (neat, cm^−1^) 3440, 2987, 2945, 1790, 1766, 1655, 1606, 1504, 1469, 1417, 1358, 1336, 1257, 1232, 1190, 1153, 1107, 991, 904, 831, 791, 715, 588. UV-Vis (EtOH) λ_max_/nm (lg ε): 291 (4.34), 220 (4.02), 204 (4.06). HRMS calcd for C_11_H_9_O_1_N_2_F_4_: 261.0646. Found 261.0643.

### 4.4. Synthesis of Complexes [Cu(hfac)_2_(***3a***)_2_], [Cu(hfac)_2_(**3b**)_2_] and [Cu(hfac)_2_(***3b***)_2_] (General Procedure)

Cu(hfac)_2_·H_2_O (248 mg, 0.5 mmol) was added to a solution of radical **3a**,**b** or amine **2b** (1.0 mmol) in CHCl_3_ (10 mL). The reaction mixture was stirred for 30 min and then was incubated at −15 °C for 10 h. The solution was filtered and evaporated. The residue was dissolved in n-hexane (5 mL); the solution was filtered and incubated at −15 °C for 10 h to prepare crystals that were filtered off and air dried.

*trans*-Bis(1,1,1,5,5,5-hexafluoropentane-2,4-dionato-*κ*^2^*O*,*O*′)bis{4-(*N*-*tert*-butyl(oxyl)amino)heptafluorotoluene-*κO*}copper (II) ([Cu(hfac)_2_(**3a**)_2_]): Brown crystals; yield 30% (163 mg). Mp 123.6 °C (DSC), mp 137.8–138.6 °C (Melting Point Apparatus). IR (KBr, cm^−1^) 3140, 2997, 1641, 1604, 1558, 1529, 1504, 1485, 1406, 1362, 1336, 1263, 1211, 1173, 1147, 1107, 1030, 989, 903, 827, 800, 746, 715, 683, 596, 530, 453. Anal. Calcd for C_32_H_20_CuF_26_N_2_O_6_: C, 35.39; H, 1.86; F, 45.48; N, 2.58. Found C, 35.07; H, 2.04; F, 45.54; N, 2.45. The molecular and crystal structures of [Cu(hfac)_2_(**3a**)_2_] were refined from single-crystal XRD data (see main text).

*trans*-Bis(1,1,1,5,5,5-hexafluoropentane-2,4-dionato-*κ*^2^*O*,*O*′)bis{4-(*N*-*tert*-butyl(oxyl)amino)tetrafluorobenzonitrile-*κO*}copper (II) ([Cu(hfac)_2_(**3b**)_2_]): Brown crystals; yield 30% (151 mg). Mp 68.8 °C (DSC), mp 70.6 °C with decomposition (Melting Point Apparatus). IR (KBr, cm^−1^) 3431, 3149, 2993, 2927, 2249, 1643, 1606, 1560, 1531, 1500, 1487, 1402, 1354, 1311, 1257, 1213, 1151, 1109, 993, 976, 831, 804, 746, 681, 596, 530, 513. Anal. Calcd for C_32_H_20_CuF_20_N_4_O_6_: C, 38.43; H, 2.02; F, 38.00; N, 5.60. Found C, 38.87; H, 2.03; F, 38.05; N, 5.60. The molecular and crystal structures of [Cu(hfac)_2_(**3b**)_2_] were refined from single-crystal XRD data (see main text).

*trans*-Bis(1,1,1,5,5,5-hexafluoropentane-2,4-dionato-*κ*^2^*O*,*O*′)bis{4-(N-tert-butyl(oxyl)amino)tetrafluorobenzonitrile-*κN*}copper (II) [Cu(hfac)_2_(**2b**)_2_]: Green crystals; yield 89% (431 mg). Mp 120.7 °C (Melting Point Apparatus). IR (KBr, cm^−1^) 3427, 31612980, 2623, 2239, 1657, 1643, 1612, 1562, 1533, 1512, 1487, 1443, 1402, 1373, 1356, 1306, 1259, 1217, 1200, 1153, 1111, 1047, 997, 982, 806, 681, 596, 528, 490. Anal. Calcd for C_33_H_22_CuF_20_N_4_O_4_: C, 39.62; H, 2.29; F, 39.17; N, 5.57. Found C, 39.55; H, 2.30; F, 39.11; N, 5.75. The molecular and crystal structures of [Cu(hfac)_2_(**2b**)_2_] were refined from single-crystal XRD data (see main text and SI).

### 4.5. X-Band ESR Measurements

ESR spectra were acquired in diluted and oxygen-free chloroform solutions at 295 K at the concentrations of ~10^−4^ M by means of the commercial Bruker X Band (9 GHz) spectrometer Elexsys E 540 (Bruker Corporation, Billerica, MA, USA). For determining the isotropic g-factors (g_iso_), we recorded X-band continuous-wave ESR spectra of a mixture of the investigated radical with the Finland trityl. Then, the known g_iso_ of the Finland trityl was used for the spectrum simulation, and the target g_iso_ value was excluded. Simulations of the solution ESR lines were carried out in software Easy Spin, which is available at http://www.easypin.org.

### 4.6. Cyclic Voltammetry Measurements

The analysis of electrochemical behavior of **3a**,**b**, [Cu(hfac)_2_(**3a**)_2_], and [Cu(hfac)_2_(**3b**)_2_] was performed in a CH_2_Cl_2_ solution by computer-controlled P-8 nano potentiostat (Elins, Chernogolovka, Russia) in combination with a three-electrode cell (Gamry, Warminster, PA, USA); 0.1 M tetrabutylammonium hexafluorophosphate served as a supporting electrolyte. Pt, a Pt wire, and Ag/AgCl were used as working, counter, and reference electrodes, respectively. The reference electrode was calibrated by measuring the redox potential of ferrocene. The scan rate was 100 mV/s.

### 4.7. Crystallographic Analysis

XRD data were collected at 200(2) K for **2b** and **3a**, and at room temperature for [Cu(hfac)_2_(**2b**)_2_], [Cu(hfac)_2_(**3a**)_2_], and [Cu(hfac)_2_(**3b**)_2_] on a Bruker Kappa Apex II CCD diffractometer using φ, ω scans of narrow frames with Mo Kα radiation (λ = 0.71073 Å) and a graphite monochromator. Absorption corrections were applied empirically using SADABS programs [49]. The structures were solved by direct methods and refined by full-matrix least-squares method against all F^2^ in anisotropic approximation (beside the atoms H) using the SHELX-97 programs set [50]. The H atoms positions, except the amino hydrogen in **2b,** were treated with the riding model. The amino hydrogen position of **2b** was localized from difference map and refined independently as well as thermal parameter. The analysis of the geometry and the intermolecular interactions for nitroxide **3a** and complexes [Cu(hfac)_2_(**3a**)_2_], [Cu(hfac)_2_(**3b**)_2_] was performed using PLATON program. [51,52] CCDC 1962404–1962408 contains the supplementary crystallographic data for this paper. These data can be obtained free of charge via http://www.ccdc.cam.ac.uk/cgi-bin/catreq.cgi, or from the Cambridge Crystallographic Data Centre, 12 Union Road, CambridgeCB2 1EZ, UK; fax: (+44) 1223 336 033; or e-mail: *deposit@ccdc.cam.ac.uk*.

### 4.8. Thermal Analysis

Thermogravimetric analysis (TGA) and differential scanning calorimetry (DSC) measurements were performed on a NETZSCH STA 409 instrument at a heating rate of 10 K/min under He flow 30 mL/min. The onset temperature of decomposition (T_0_), the melting temperature (T_m_) were determined using NETZSCH Proteus Thermal Analysis software.

### 4.9. Magnetic Measurements

Magnetic susceptibility of the polycrystalline samples of complexes [Cu(hfac)_2_(**3a**)_2_] and [Cu(hfac)_2_(**3b**)_2_] was measured with a Quantum Design MPMSXL SQUID magnetometer in the temperature range 2 to 300 K with a magnetic field of up to 5 kOe. Diamagnetic corrections were made via the Pascal constants. The effective magnetic moment was calculated as μ_eff_(T) = [(3k/N_A_μ_B_^2^)χT]^1/2^ ≈ (8χT)^1/2^.

## Figures and Tables

**Table 1:** Half-wave potentials for the studied compounds.

Compound	E_1/2_^ox^, V	E_1/2_^Red^, V
**3a** *	1.04	−1.44
**3b** *	1.00	−1.14
[Cu(hfac)_2_(**3a**)_2_] *	1.04	−1.40
[Cu(hfac)_2_(**3b**)_2_] *	1.06	−1.20
**4a** **	0.38	−1.63
**4b** **	0.53	−1.37

* Data obtained in a CH_2_Cl_2_/0.1 M *n*-Bu_4_NPF_6_ solution at 298 K vs. Fc/Fc^+^. ** Data obtained in a MeCN/0.1 M *n*-Bu_4_NBF_4_ solution [33].

**Table 2:** XRD data on radical **3a** and complexes [Cu(hfac)_2_(**3a**)_2_] and [Cu(hfac)_2_(**3b**)_2_].

Compound	3a	[Cu(hfac)_2_(3a)_2_]	[Cu(hfac)_2_(3b)_2_]
Empirical formula	C_11_H_9_F_7_NO	C_32_H_20_CuF_26_N_2_O_6_	C_32_H_20_CuF_20_N_4_O_6_
Formula weight	304.19	1086.04	1000.06
Crystal system	Orthorhombic	Triclinic	Monoclinic
Space group	Pbca	P-1	P*2_1_*/*c*
*a*, Å	11.6131(7)	10.0680(3)	11.3678(8)
*b*	10.9047(7)	13.8773(5)	8.6913(7)
*c*	19.7695(12)	16.1651(6)	20.219(1)
*α*, ^o^	90.00	96.289(2)	90.00
*β*, ^o^	90.00	102.367(2)	90.158(3)
*γ*, ^o^	90.00	104.872(2)	90.00
Volume, Å^3^	2503.6(3)	2099.6(1)	1997.6(2)
Z	8	2	2
Density (calcd.), mg·m^−3^	1.614	1.718	1.663
Abs. coefficient, mm^−1^	0.174	0.680	0.687
F(000)	1224	1074	994
Crystal size, mm^3^	0.40 × 0.70 × 1.00	0.30 × 0.40 × 0.50	0.10 × 0.12 × 0.80
Θ range for data collection, °	2.76–26.07	1.31–26.43	1.79–25.05
Index ranges	−14 ≤ h ≤ 14, −13 ≤ k ≤ 13, −24 ≤ l ≤ 24	−12 ≤ h ≤ 12, −17 ≤ k ≤ 17, −20 ≤ l ≤ 20	−13 ≤ h ≤ 13, −10 ≤ k ≤ 10, −24 ≤ l ≤ 24
Reflections collected	34,642	64,762	26,173
Independent reflections	2473 R(int) 0.0266	8633 R(int) 0.052	3530 R(int) 0.069
Completeness to θ 50º, %	99.6	99.7	99.7
Data/restraints/parameters	2473/0/184	8633/0/613	3530/48/376
Goodness-of-fit on *F^2^*	1.044	1.007	1.03
Reflections with *I > 2σ(I)*	2057	5535	2587
Final R indices at *I > 2σ(I)*	*R*_1_ 0.0555, *wR*_2_ 0.1399	*R*_1_ 0.0630, *wR*_2_ 0.1764	*R*_1_ 0.0530, *wR*_2_ 0.1466
Final R indices (all data)	*R*_1_ 0.680, *wR*_2_ 0.1554	*R*_1_ 0.1034, *wR*_2_ 0.2165	*R*_1_ 0.0791, *wR*_2_ 0.1716
Largest diff. peak/hole, e·Å^−3^	0.59/−0.27	0.73/−0.47	0.58/−0.38

**Table 3:** Selected geometrical parameters of complexes [Cu(hfac)_2_(**3a**)_2_] and [Cu(hfac)_2_(**3b**)_2_].

Parameter	[Cu(hfac)_2_(3a)_2_] ^1^		[Cu(hfac)_2_(3b)_2_]
	Bond Lengths, Å		
Cu1–O1	2.411(5)	2.398(4)	2.452(4)
Cu1–O2	1.942(3)	1.946(3)	1.944(2)
Cu1–O3	1.935(3)	1.935(3)	1.937(2)
	Bond angles, °		
O1–Cu1–O2	89.0(1)	88.1(1)	86.6(1)
O1–Cu1–O3	84.1(1)	82.3(1)	83.9(1)
O2–Cu1–O3	92.4(1)	92.4(1)	92.7(1)

^1^ For two independent moieties.

## Supplementary Materials

The following are available online, Figure S1: ^1^H NMR spectrum of **2a**, Figure S2: ^13^C NMR spectrum of **2a**, Figure S3: ^19^F NMR spectrum of **2a**, Figure S4: IR spectrum of **2a**, Figure S5: ^1^H NMR spectrum of **2b**, Figure S6: ^13^C NMR spectrum of **2b**, Figure S7: ^19^F NMR spectrum of **2b**, Figure S8: IR spectrum of **2b**, Figure S9: IR spectrum of **3a**, Figure S10: UV spectrum of **3a**, Figure S11: IR spectrum of **3b**, Figure S12: UV spectrum of **3b**, Figure S13: IR spectrum of [Cu(hfac)_2_(**3a**)_2_], Figure S14: IR spectrum of [Cu(hfac)_2_(**3a**)_2_] after sublimation, Figure S15: IR spectrum of [Cu(hfac)_2_(**3b**)_2_], Figure S16: IR spectrum of [Cu(hfac)_2_(**3a**)_2_] after sublimation, Figure S17: IR spectrum of [Cu(hfac)_2_(**2b**)_2_], Figure S18: Experimental and simulated ESR spectrum for **3b**, Figure S19: DSC and TG curves for [Cu(hfac)_2_(**3a**)_2_], Figure S20: DSC and TG curves for [Cu(hfac)_2_(**3b**)_2_], Figure S21: Cyclic voltammograms of nitroxides **3a**,**b** and complexes [Cu(hfac)_2_(**3a**)_2_], [Cu(hfac)_2_(**3b**)_2_] in CH_2_Cl_2_ solution, S22: Crystallographic data for amine **2b** and complex [Cu(hfac)_2_(**2b**)_2_], Figure S22: The molecular structure and atom-labelling, the fragment of crystal packing of compound **2b**.
