# Supplementary material for: Aromatic SNF-Approach to Fluorinated Phenyl tert-Butyl Nitroxides
Source: Molecules. 2019 Dec 8;24(24):4493. doi: 10.3390/molecules24244493 (PMC6943699; doi:10.3390/molecules24244493)
Supplement: Supplementary file 1 [file molecules-24-04493-s001.pdf]

Supporting Information

Aromatic S_N^F-Approach to Fluorinated Phenyl *tert*-Butyl Nitroxides

Evgeny Tretyakov [1,2,*], Pavel Fedyushin [1], Elena Panteleeva [1,2], Larisa Gurskaya [1], Tatyana Rybalova [1,2], Artem Bogomyakov [2,3], Elena Zaytseva [1,2], Maxim Kazantsev [1], Inna Shundrina [1,2] and Victor Ovcharenko [3,*]

[1] N. N. Vorozhtsov Institute of Organic Chemistry, 9 Ac. Lavrentiev Avenue, Novosibirsk 630090, Russia; feduyshin@nioch.nsc.ru (P.F); pantel@nioch.nsc.ru (E.P.); gurlar82@nioch.nsc.ru (L.G.); rybalova@nioch.nsc.ru (T.R.); elena@nioch.nsc.ru (E.Z.); kazancev@nioch.nsc.ru (M.K.); ishund@nioch.nsc.ru (I.S.)
[2] Novosibirsk State University, 2 Pirogova Str., Novosibirsk 630090, Russia;
[3] International Tomography Center, 3a Institutskaya Str., Novosibirsk 630090, Russia; bus@tomo.nsc.ru (A.B.)
* Correspondence: tretyakov@nioch.nsc.ru (E.T.); ovchar@tomo.nsc.ru (V.O.)

Content:

NMR, IR and electronic spectroscopy data S2
ESR spectroscopy data S19
DSC and TG data for complexes [Cu(hfac)$_2$(**3a**)$_2$], [Cu(hfac)$_2$(**3b**)$_2$] S20
CVA data for nitroxides **3a,b** and complexes [Cu(hfac)$_2$(**3a**)$_2$], [Cu(hfac)$_2$(**3b**)$_2$] S21
Crystallographic data for amine **2b** and complex [Cu(hfac)$_2$(**2b**)$_2$] S22

NMR, IR and electronic spectroscopy data

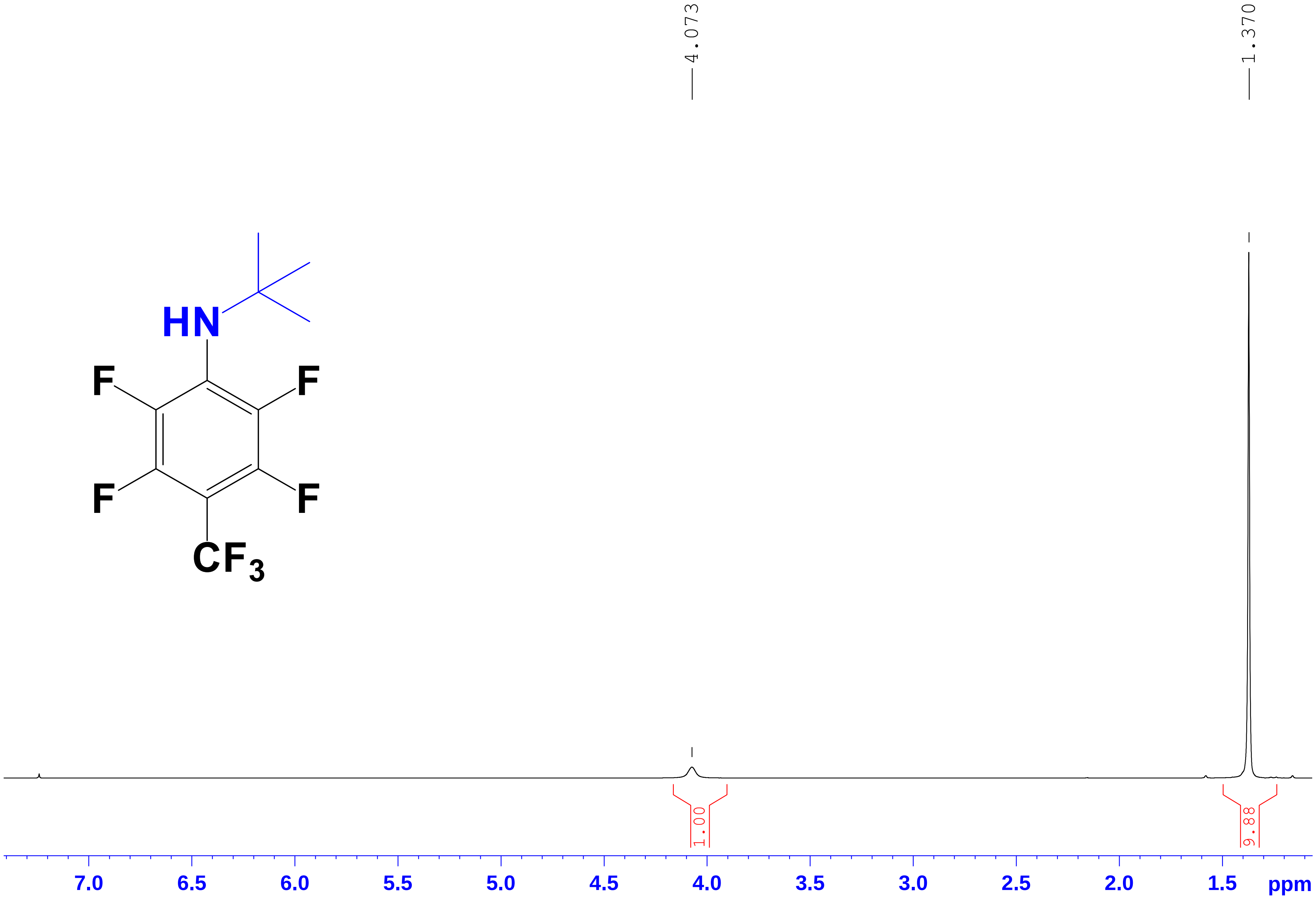

Figure S1. ^{1}H NMR spectrum of **2a** (300.13 MHz, $CDCl_3$).

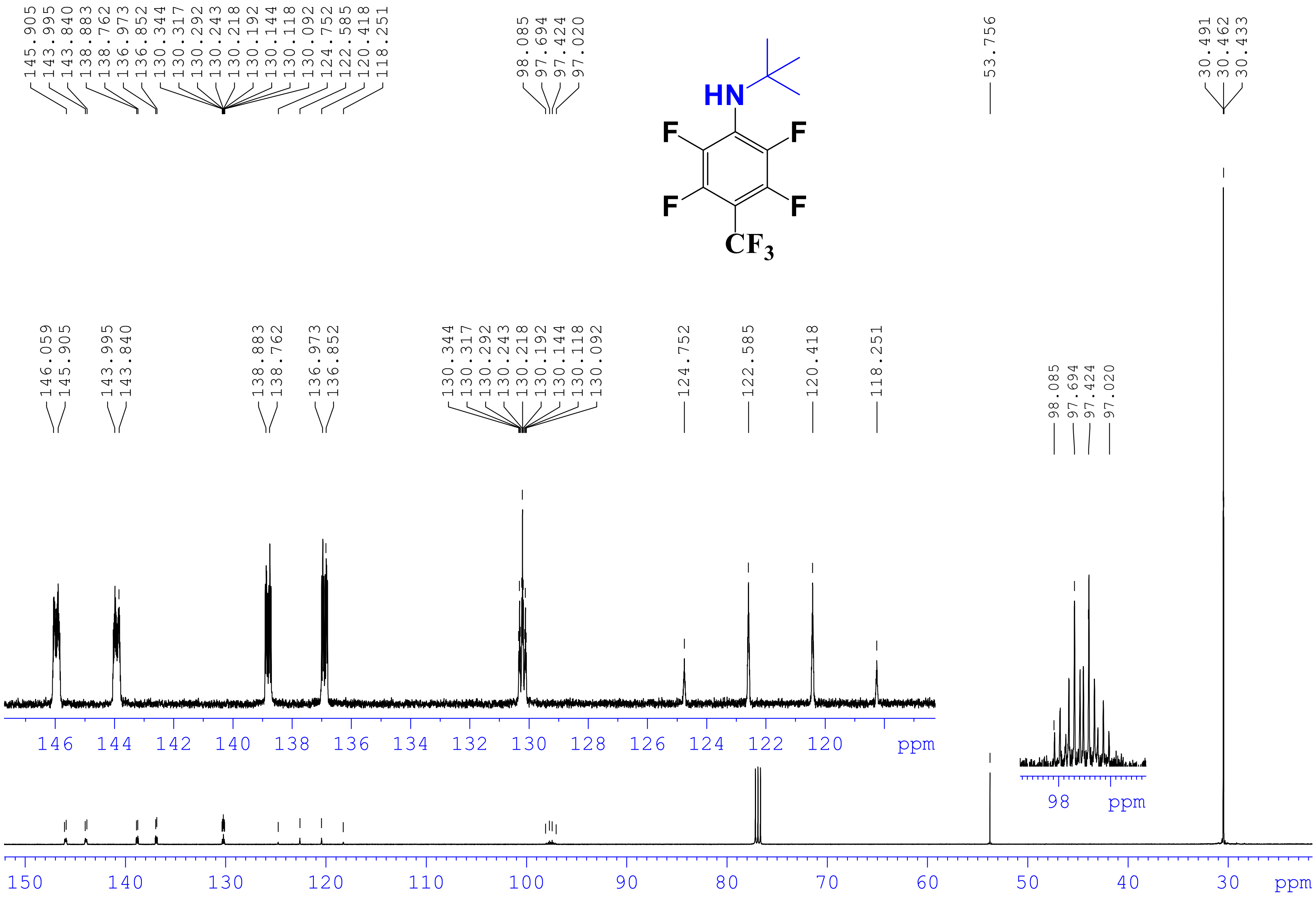

Figure S2. ^{13}C NMR spectrum of **2a** (125.75 MHz, $CDCl_3$).

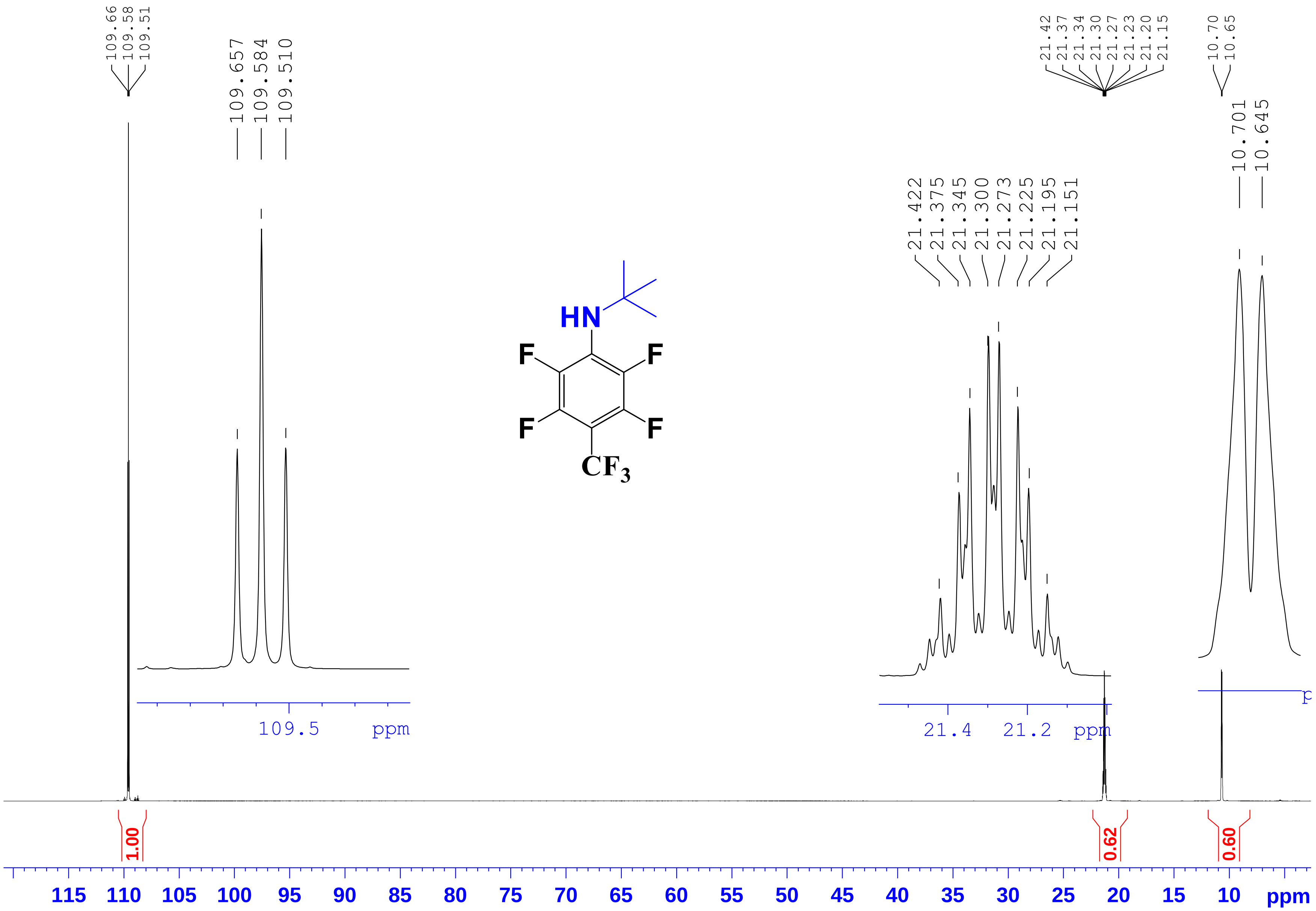

Figure S3. ^{19}F NMR spectrum of **2a** (282.37 MHz, $CDCl_3$).

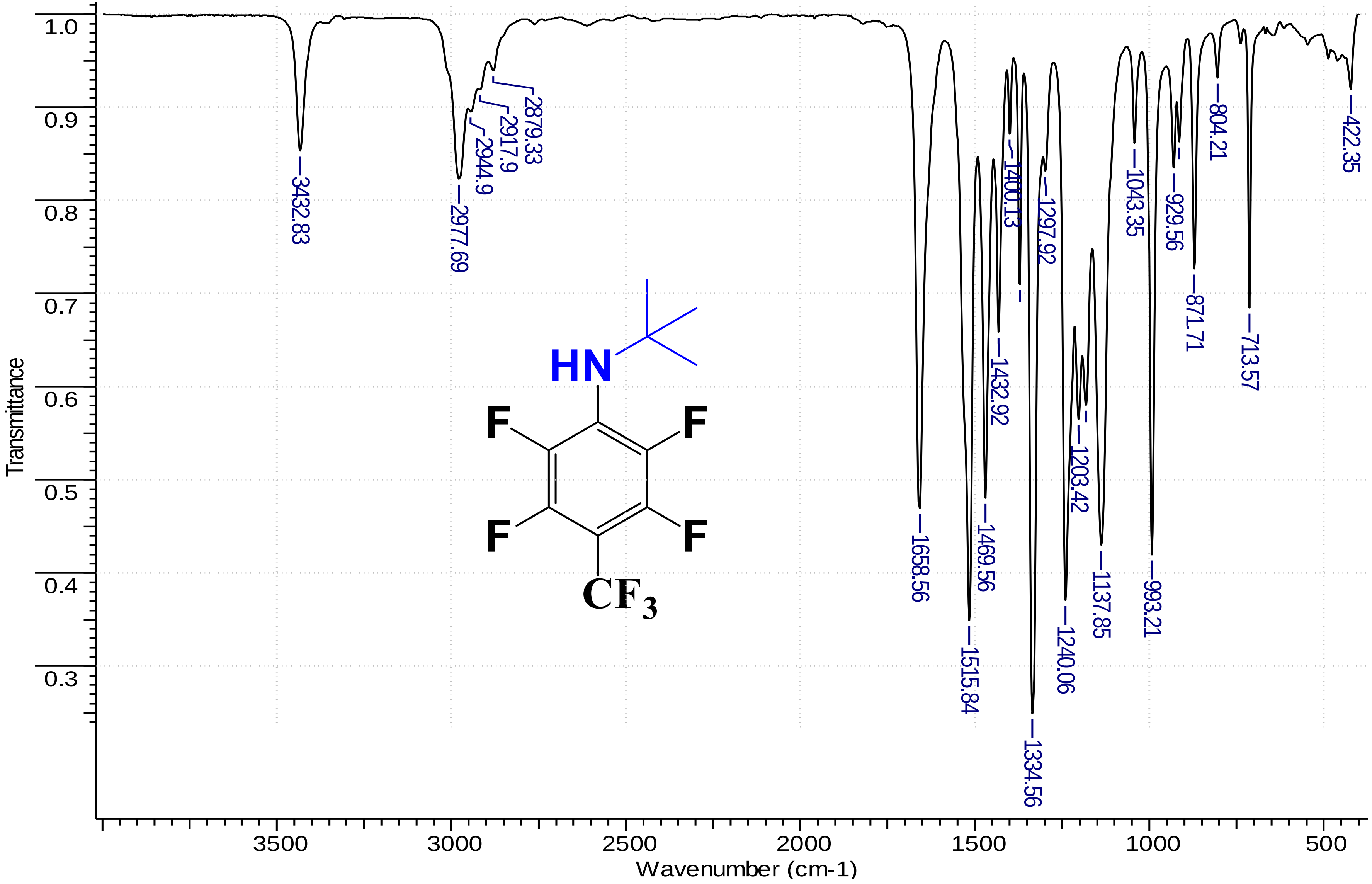

Figure S4. IR spectrum of **2a** (KBr).

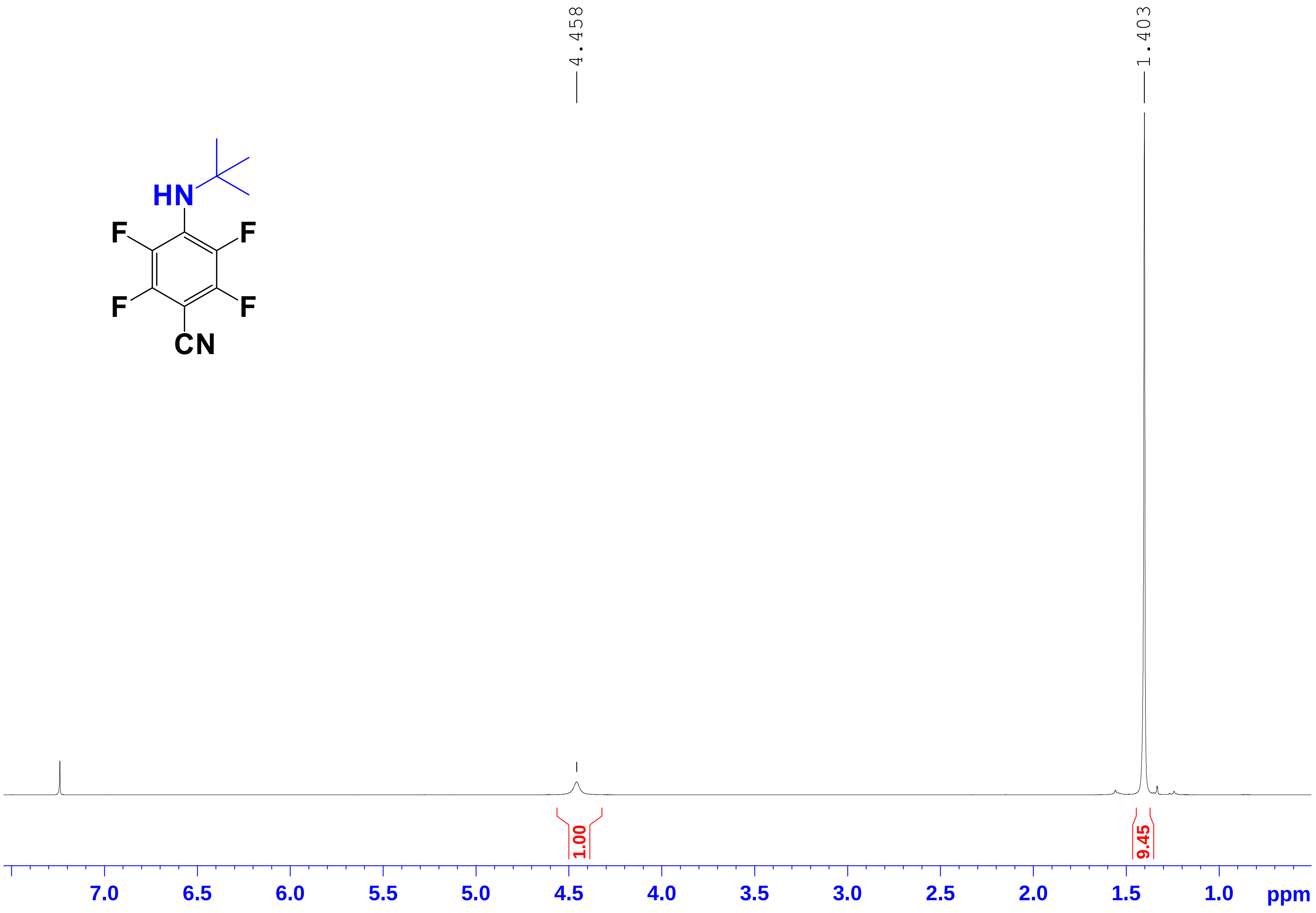

Figure S5. ^{1}H NMR spectrum of **2b** (400.13 MHz, $CDCl_3$).

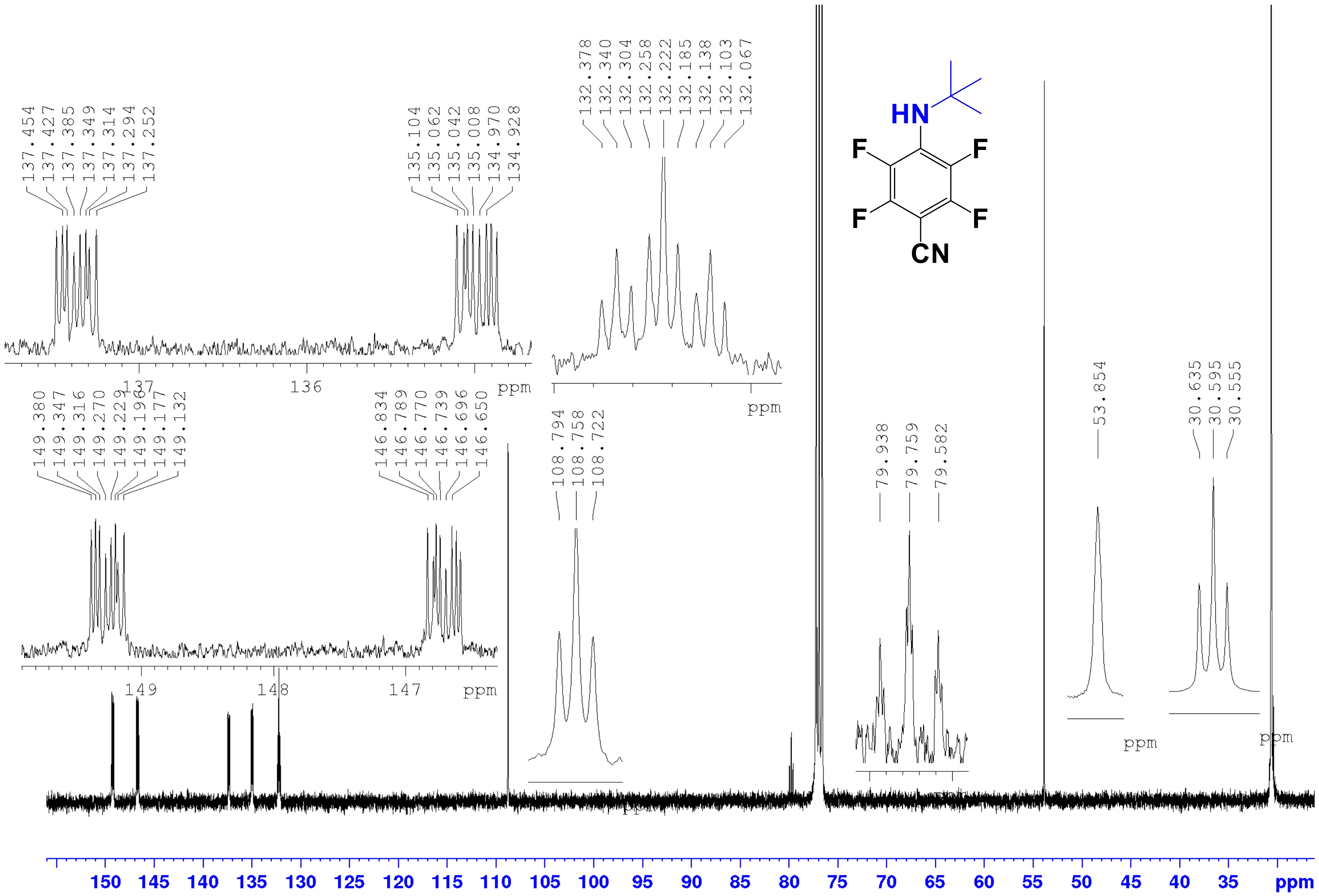

Figure S6. ^{13}C NMR spectrum of **2b** (100.62 MHz, $CDCl_3$).

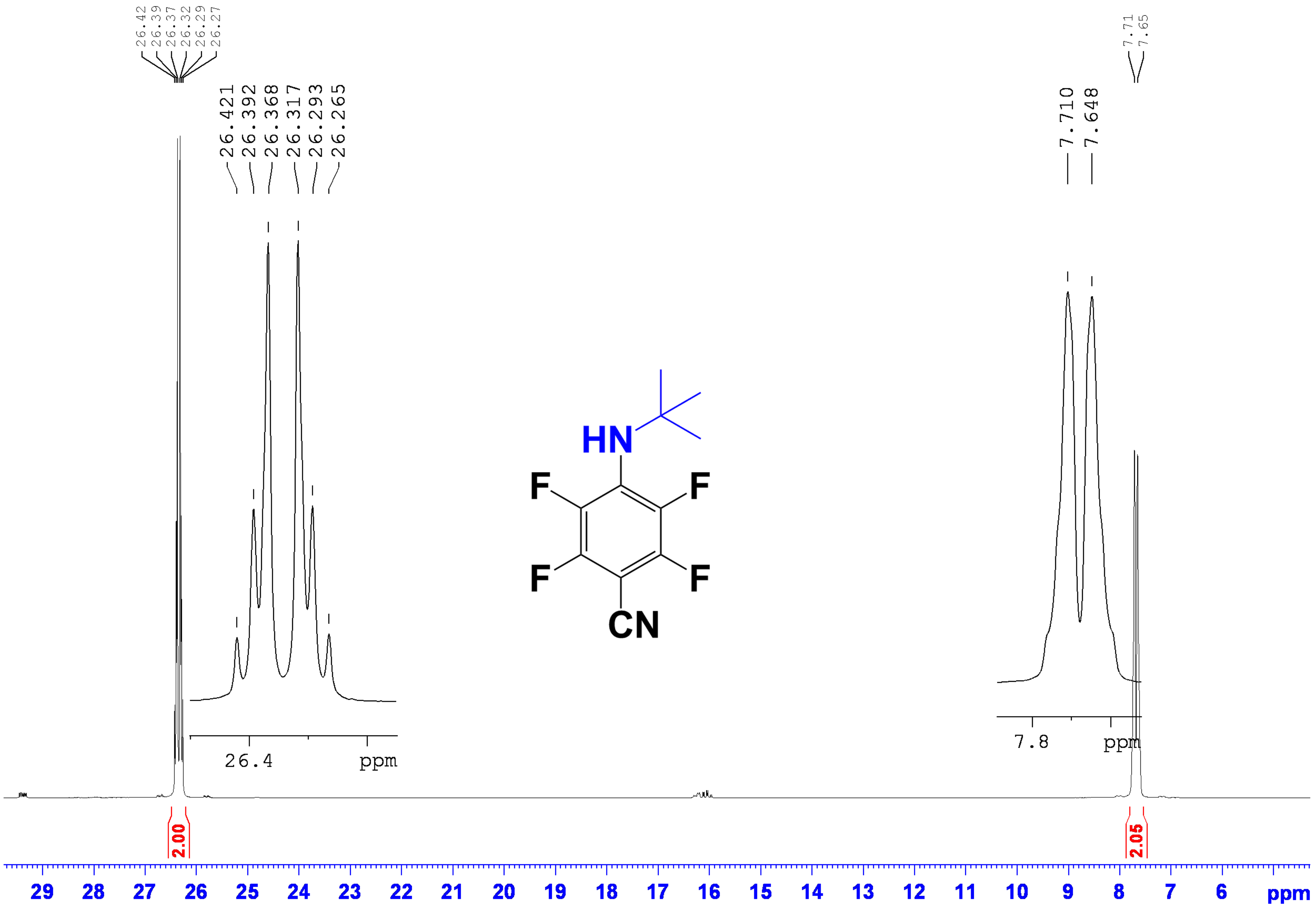

Figure S7. ^{19}F NMR spectrum of **2b** (282.37 MHz, $CDCl_3$).

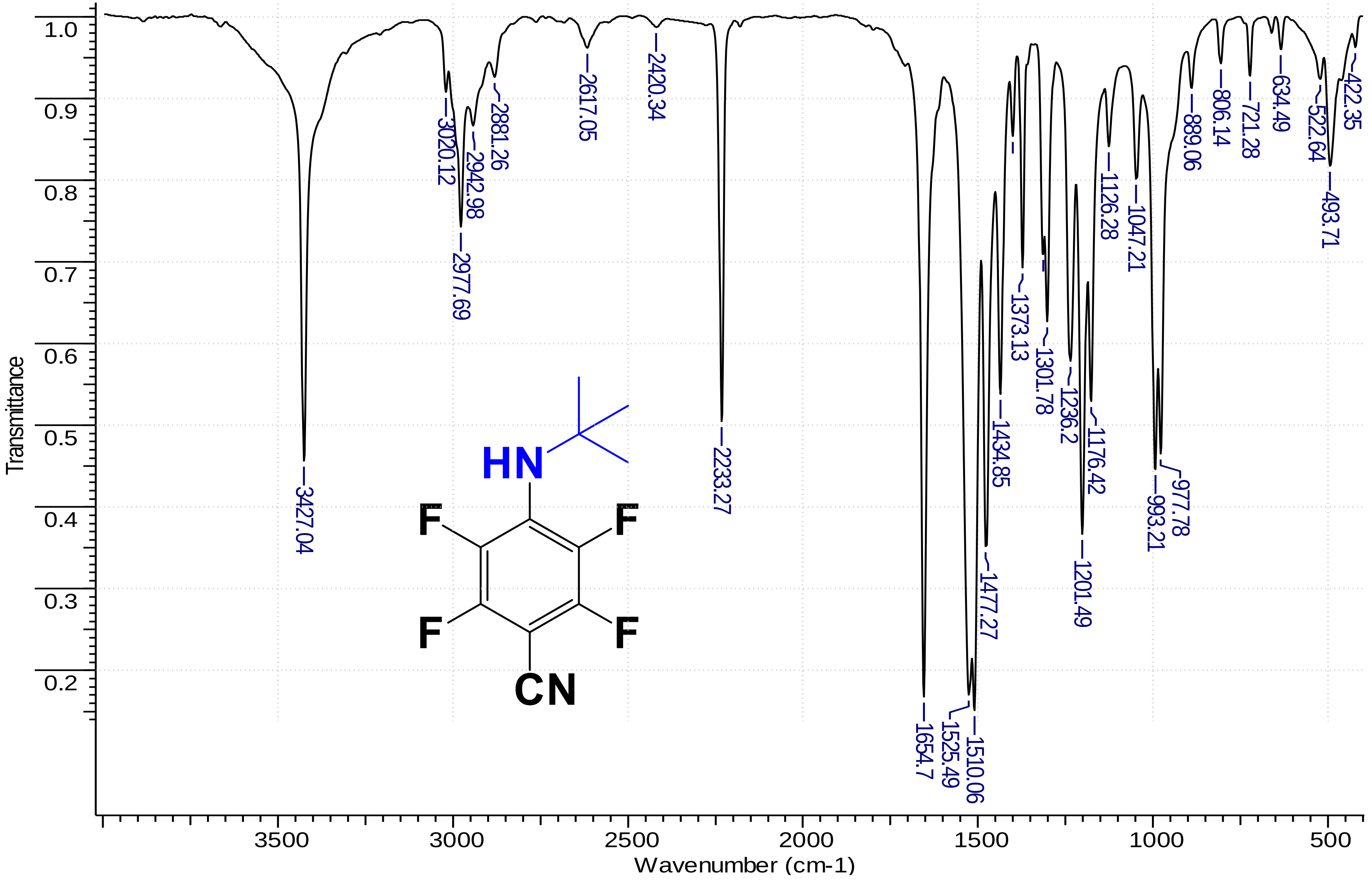

Figure S8. IR spectrum of **2b** (KBr).

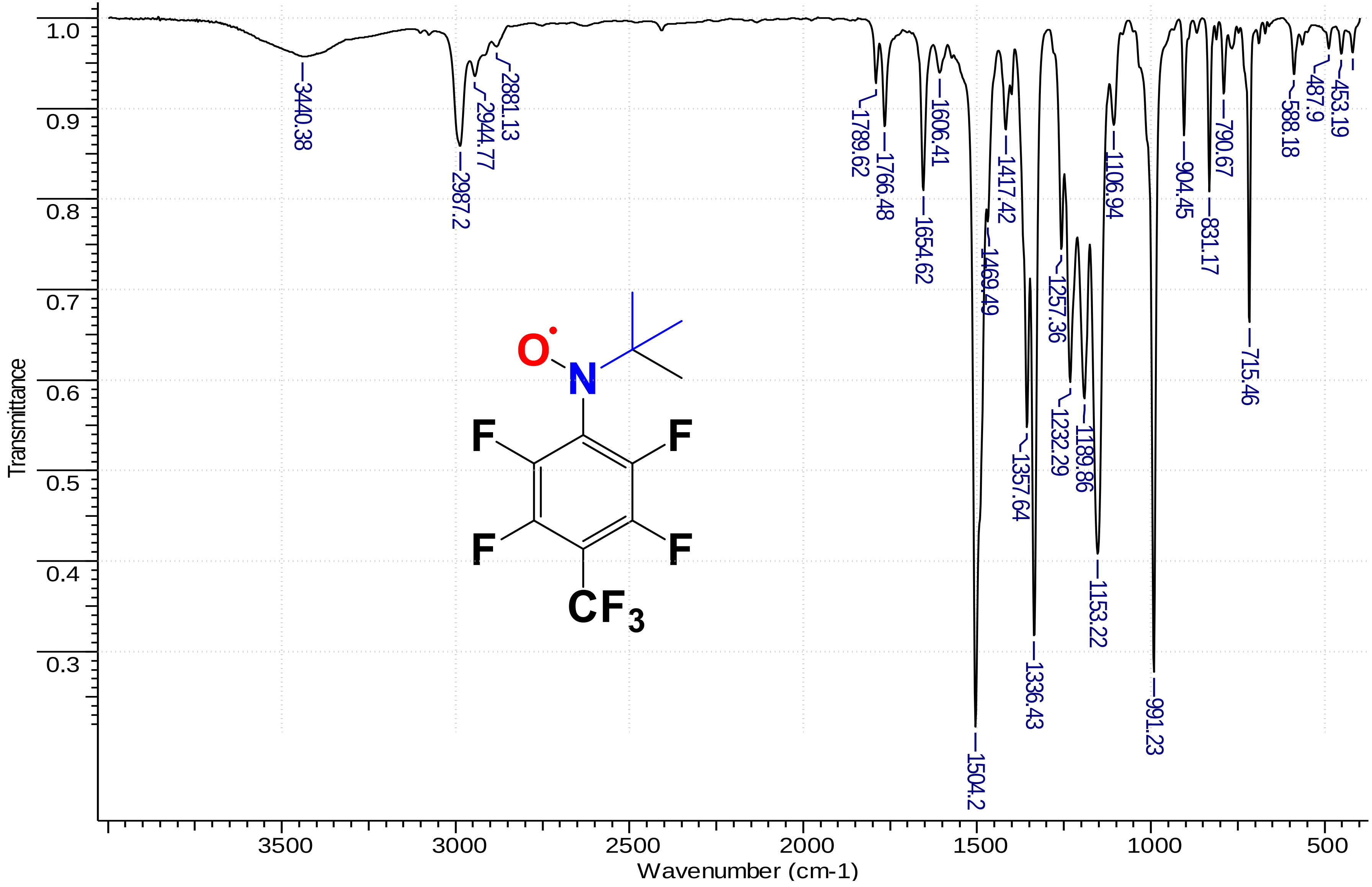

Figure S9. IR spectrum of **3a** (KBr).

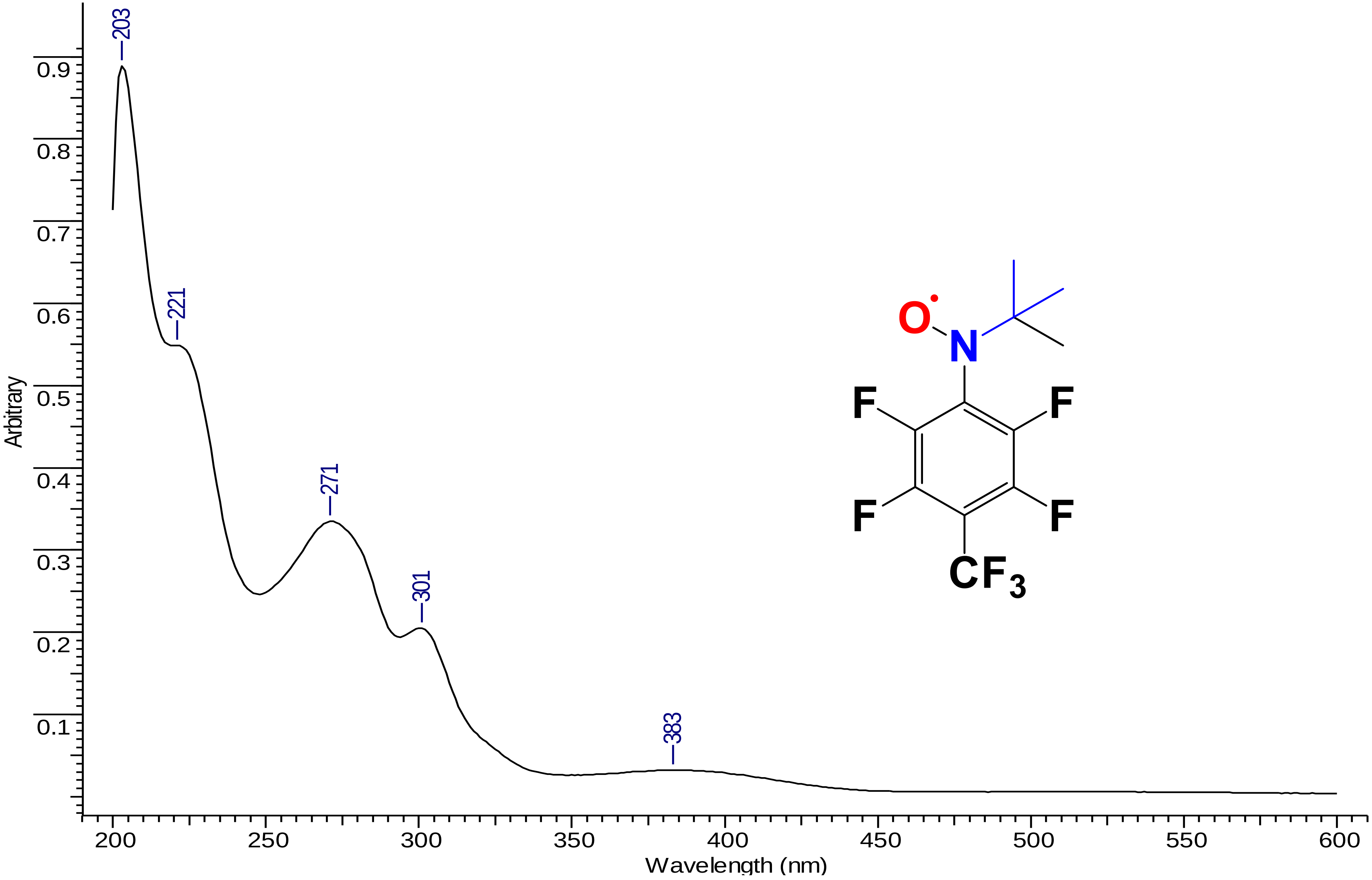

Figure S10. UV spectrum of **3a** (EtOH).

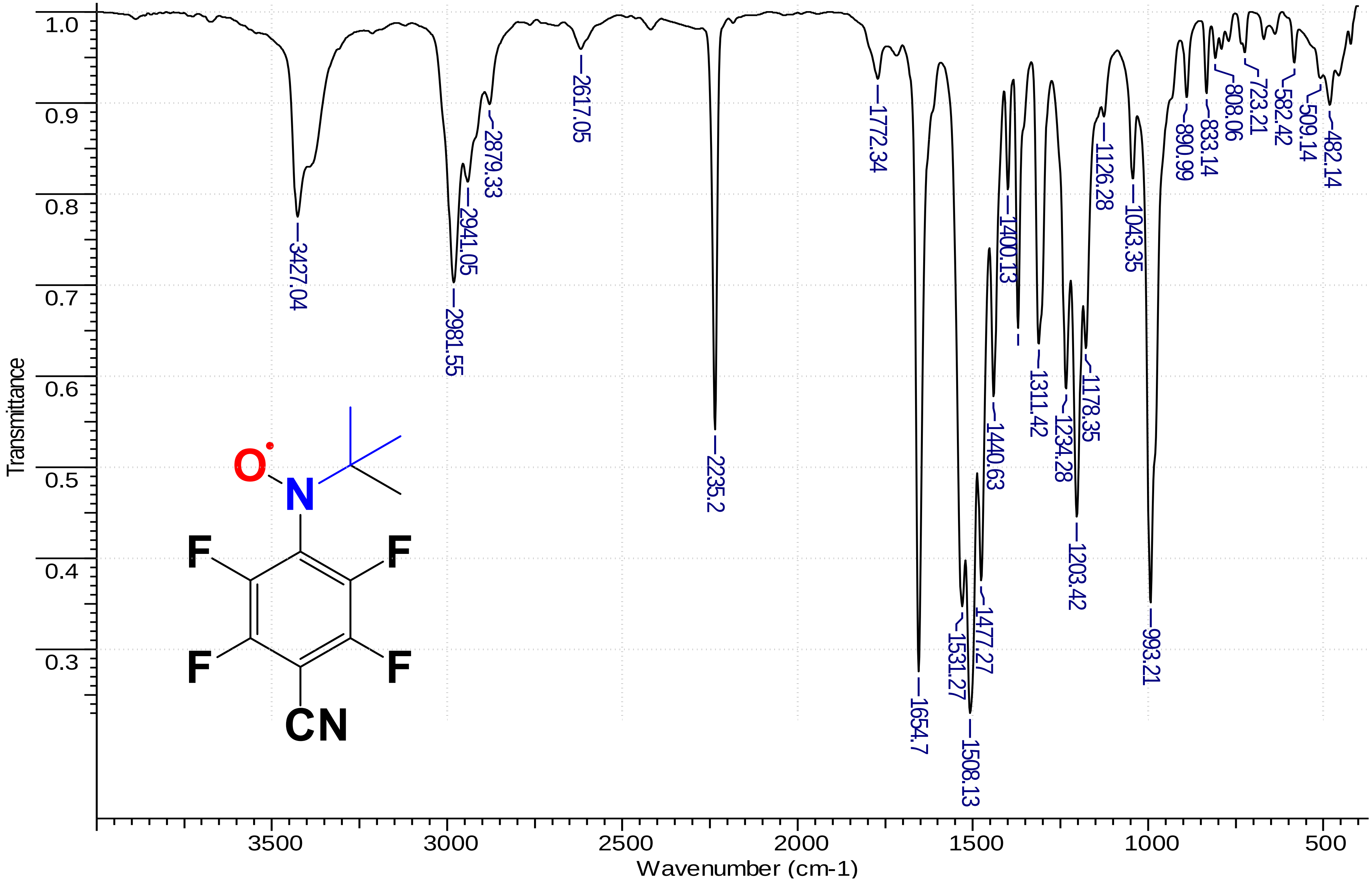

Figure S11. IR spectrum of **3b** (neat).

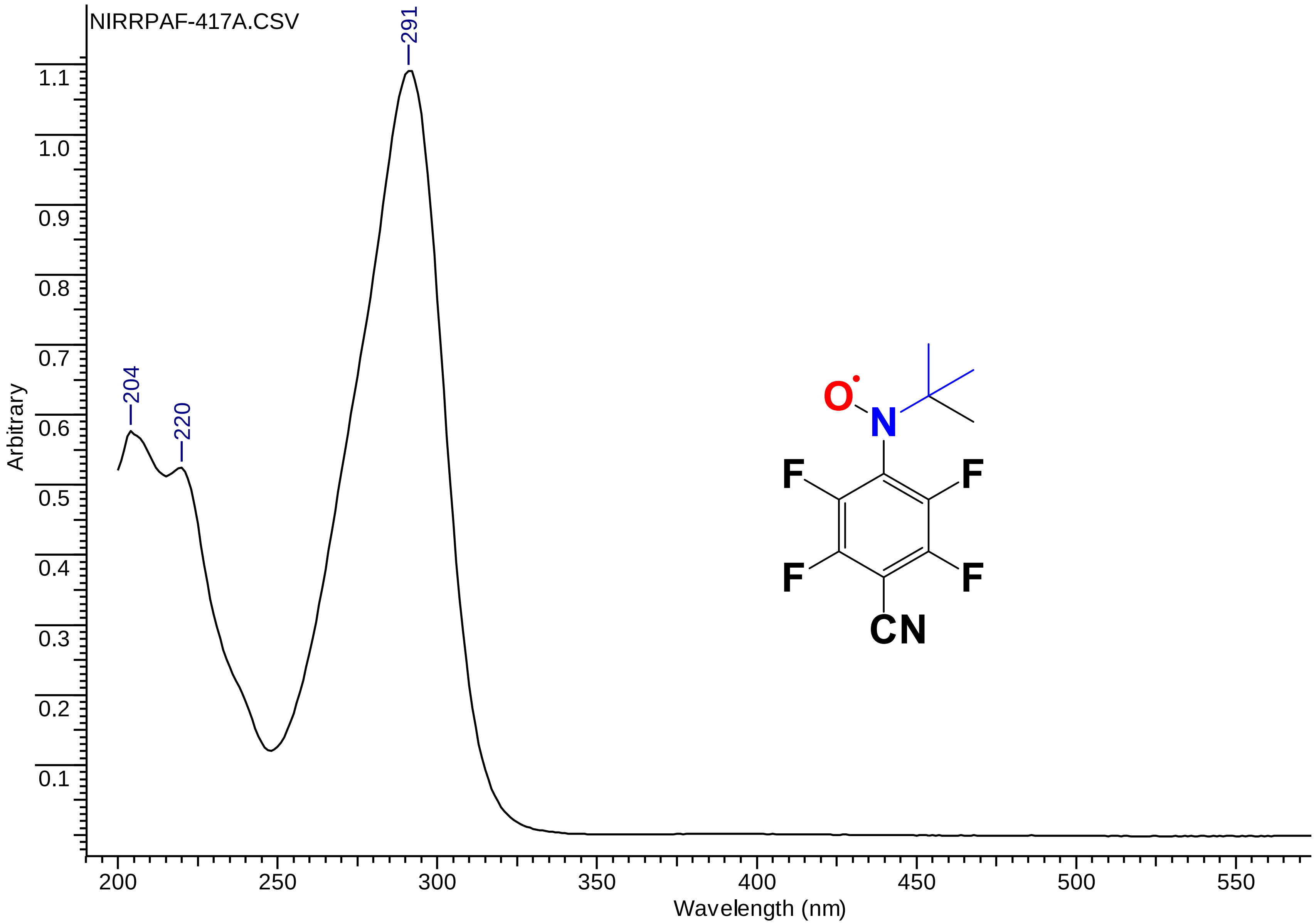

Figure S12. UV spectrum of **3b** (EtOH).

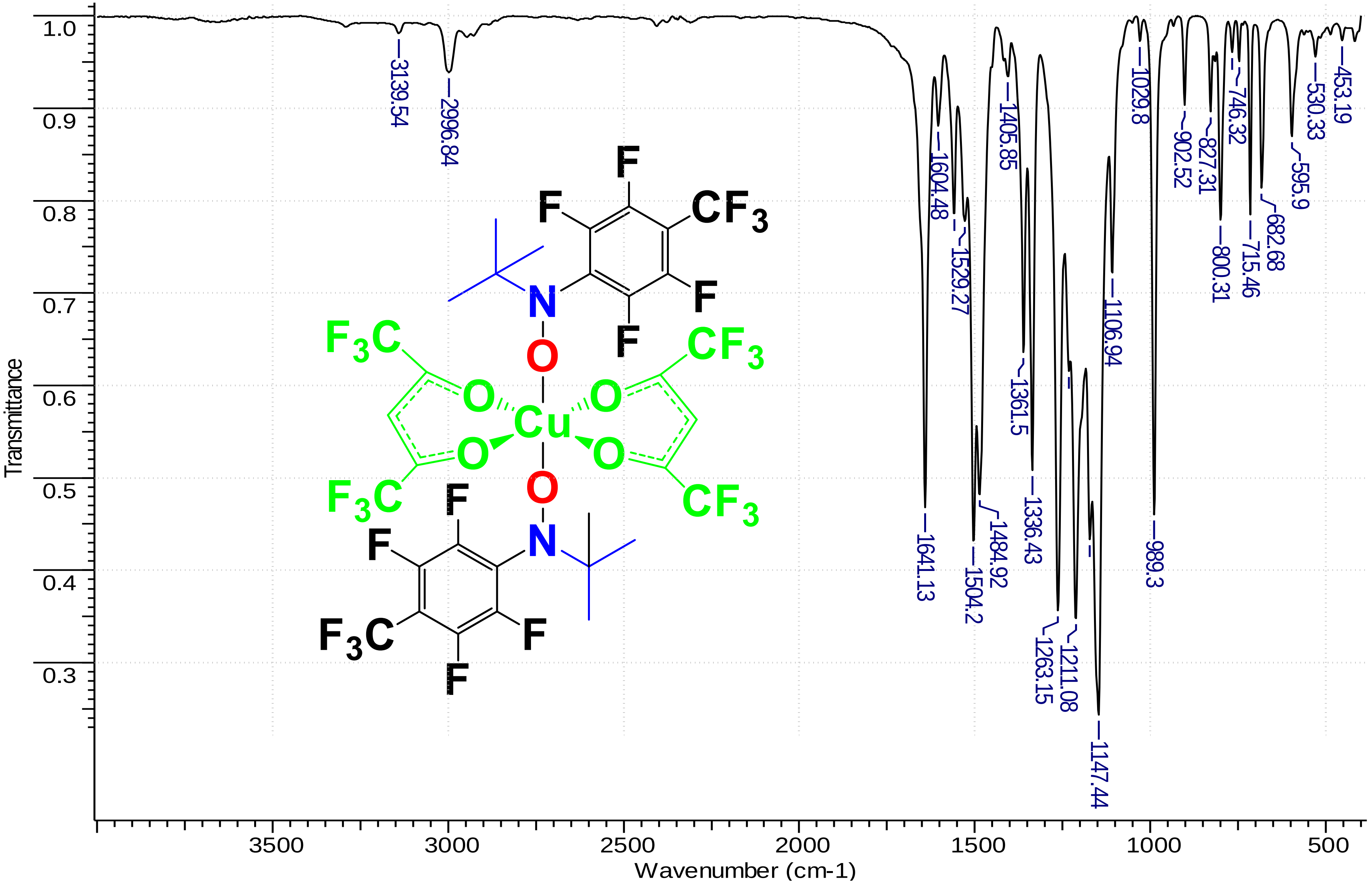

Figure S13. IR spectrum of [Cu(hfac)$_2$(**3a**)$_2$] (KBr).

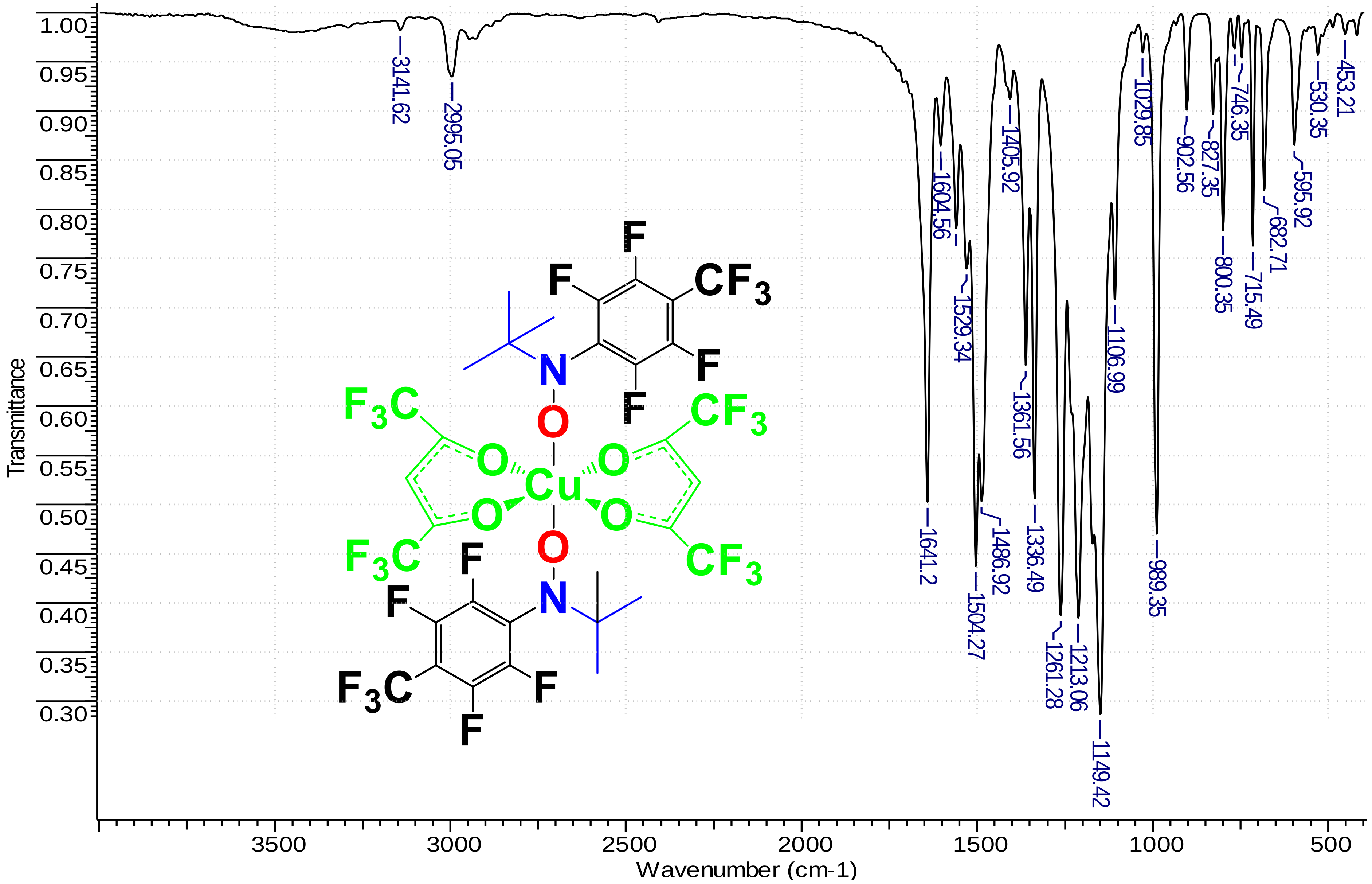

Figure S14. IR spectrum of [Cu(hfac)$_2$(**3a**)$_2$] (KBr) after sublimation.

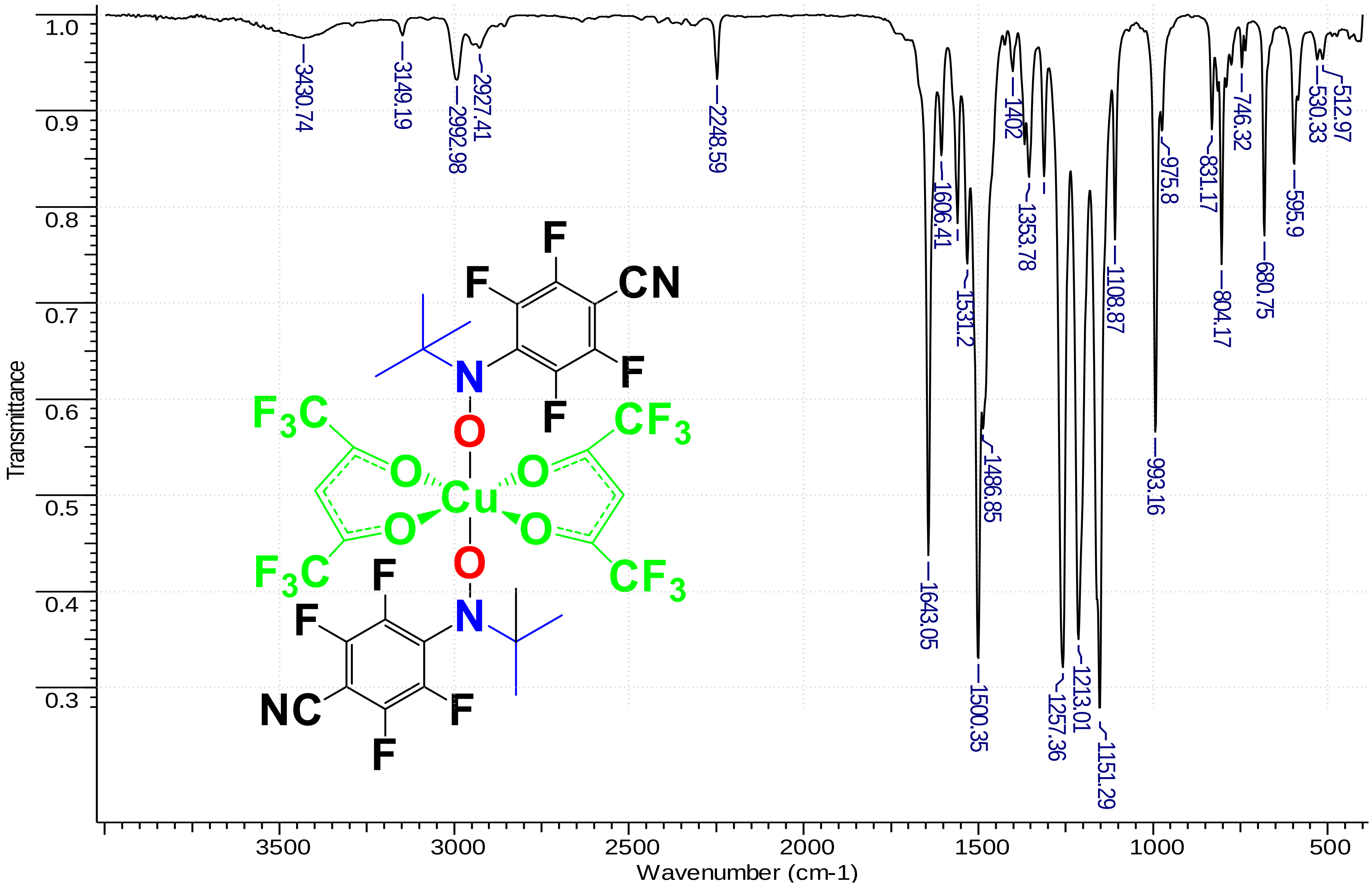

Figure S15. IR spectrum of [Cu(hfac)$_2$(**3b**)$_2$] (KBr).

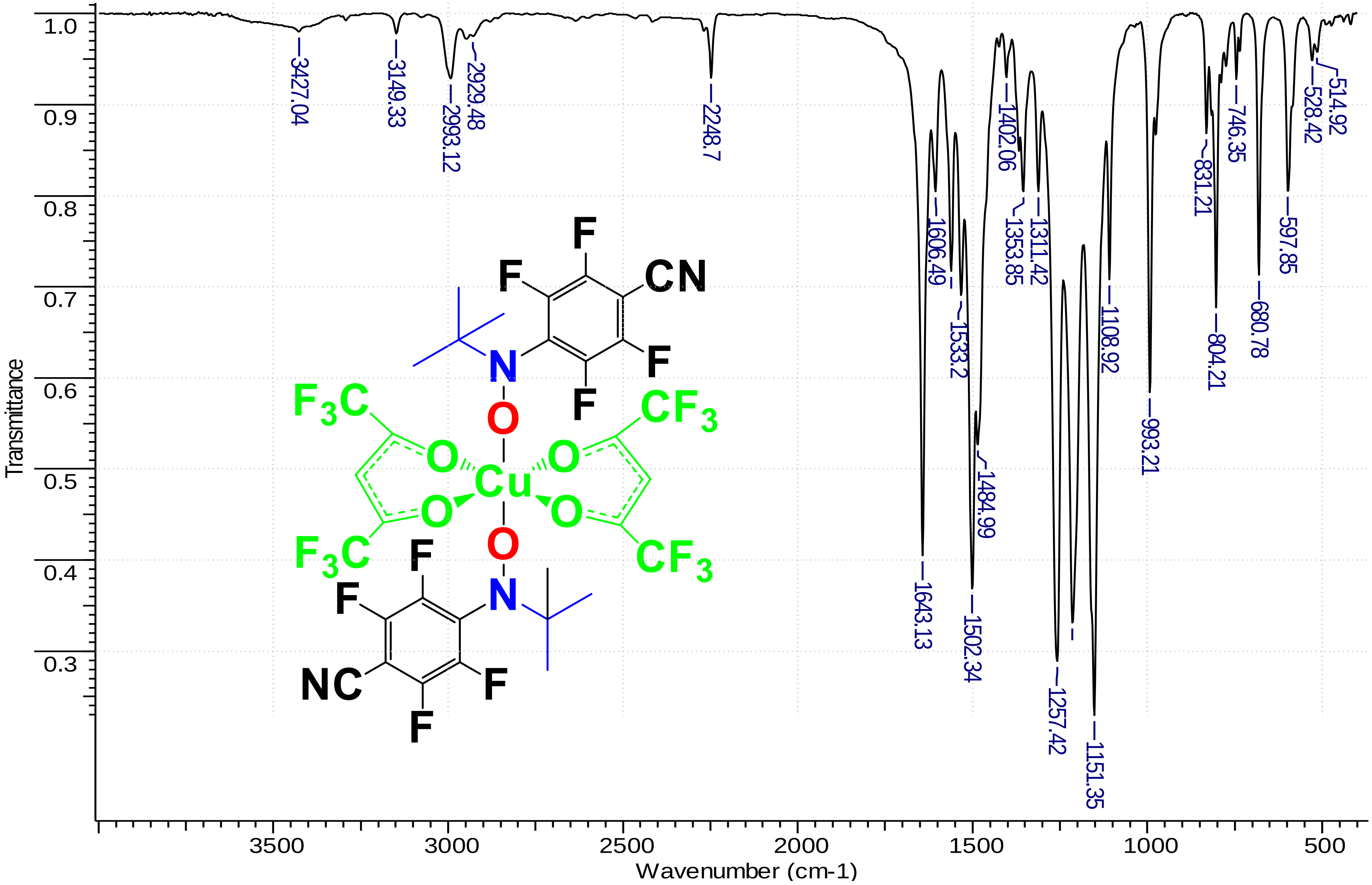

Figure S16. IR spectrum of [Cu(hfac)$_2$(**3b**)$_2$] (KBr) after sublimation.

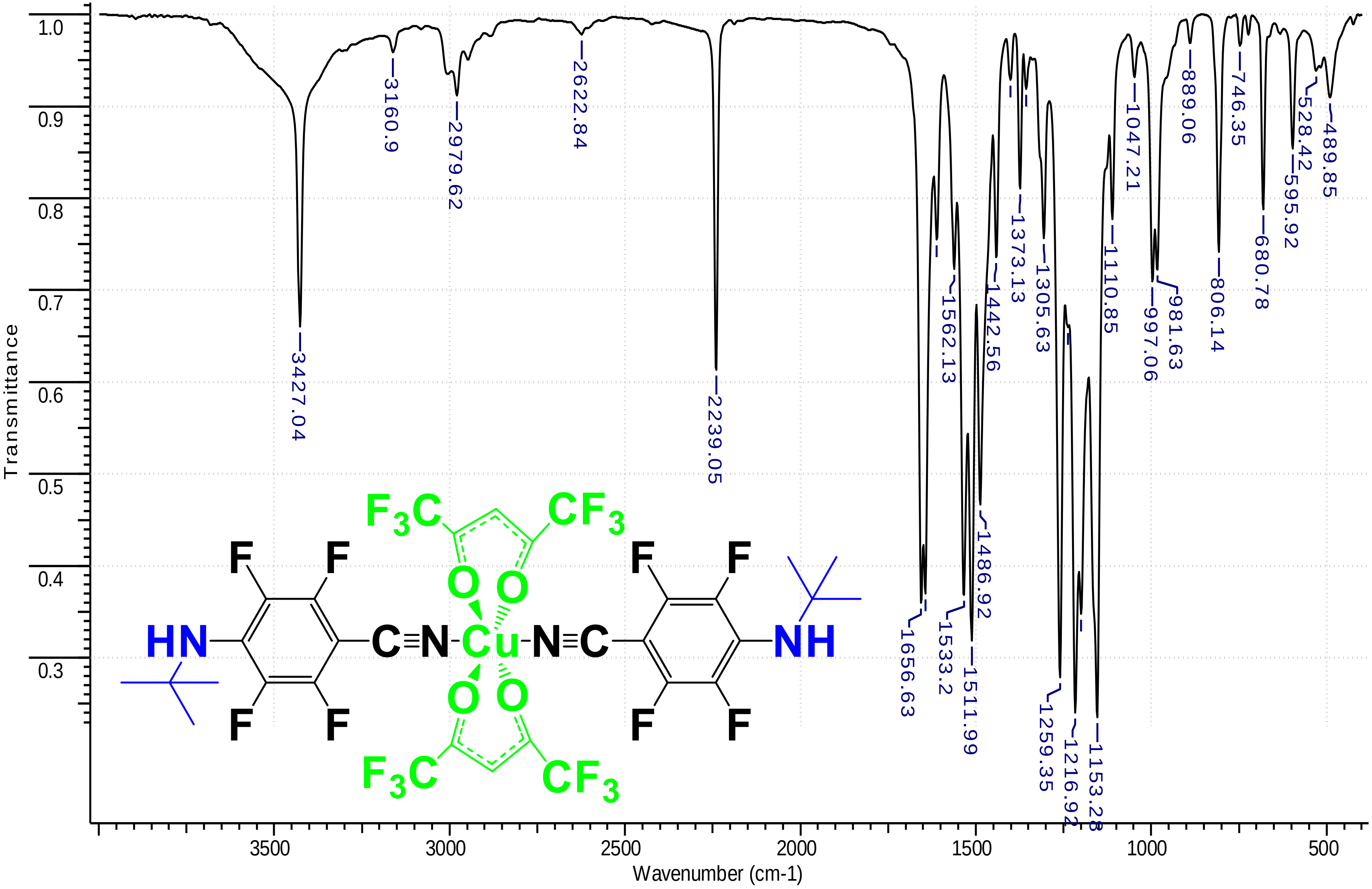

Figure S17. IR spectrum of [Cu(hfac)$_2$(**2b**)$_2$] (KBr).

ESR spectroscopy data

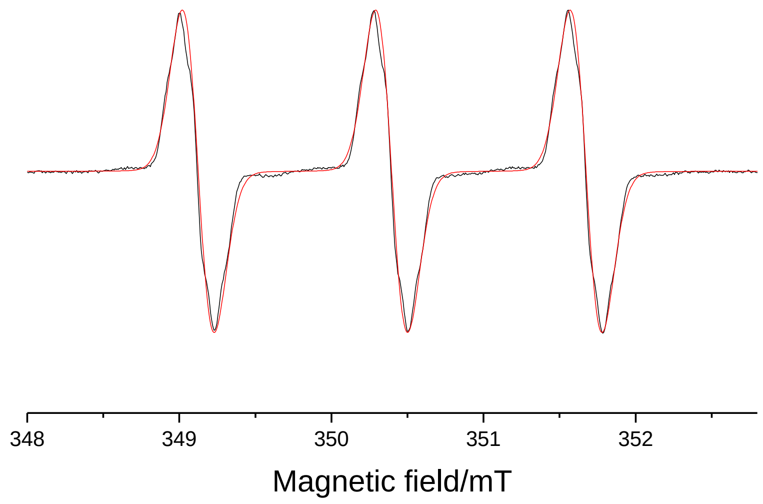

Figure S18. Experimental (black curve) and simulated (red curve) ESR spectrum for **3b**.

DSC and TG data for complexes [Cu(hfac)$_2$(3a)$_2$], [Cu(hfac)$_2$(3b)$_2$]

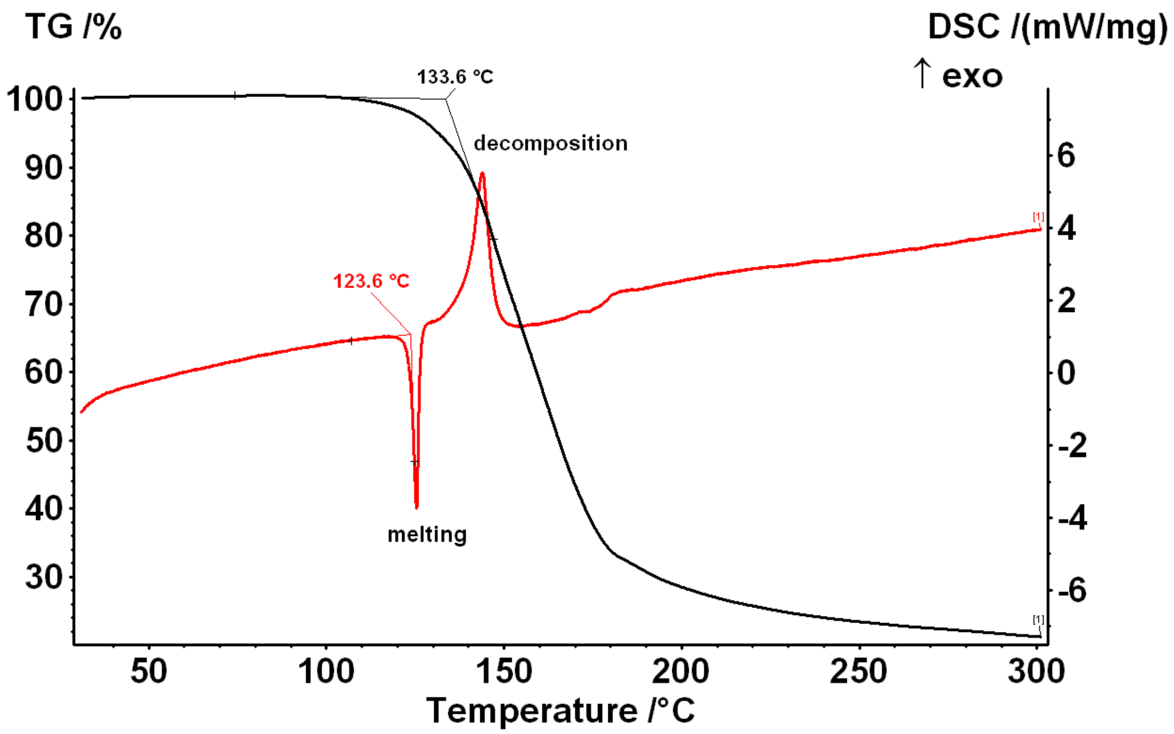

Figure S19. DSC and TG curves for [Cu(hfac)$_2$(**3a**)$_2$].

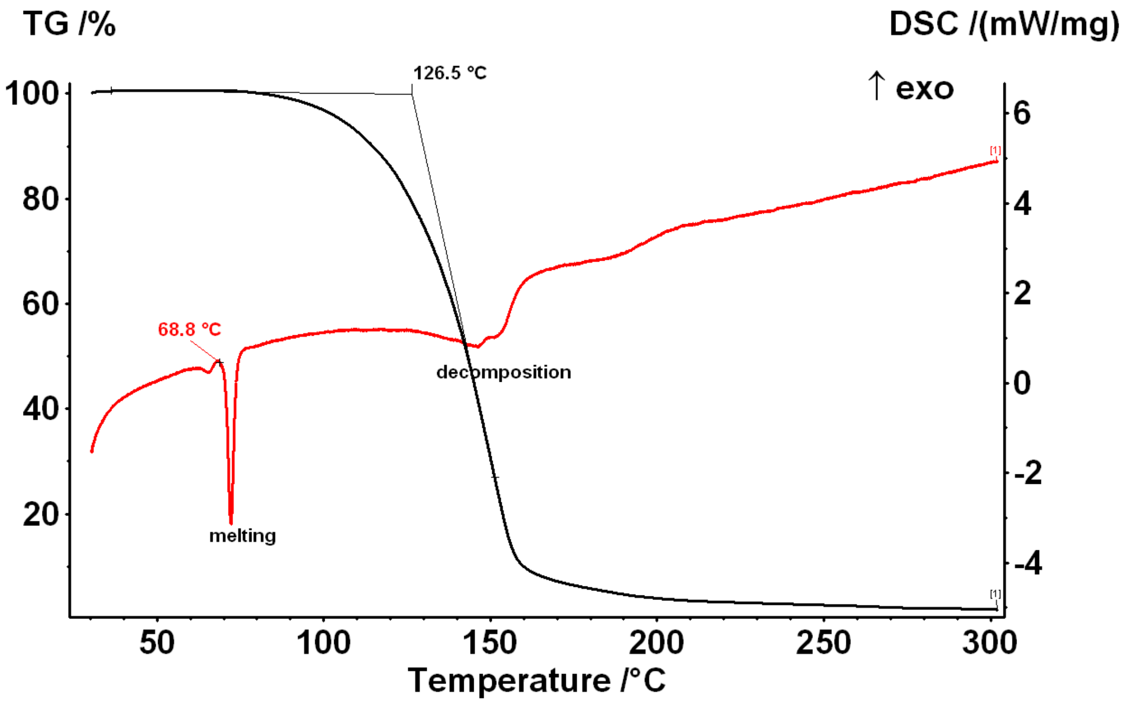

Figure S20. DSC and TG curves for [Cu(hfac)$_2$(**3b**)$_2$].

CVA data for nitroxides 3a,b and complexes [Cu(hfac)$_2$(3a)$_2$], [Cu(hfac)$_2$(3b)$_2$]

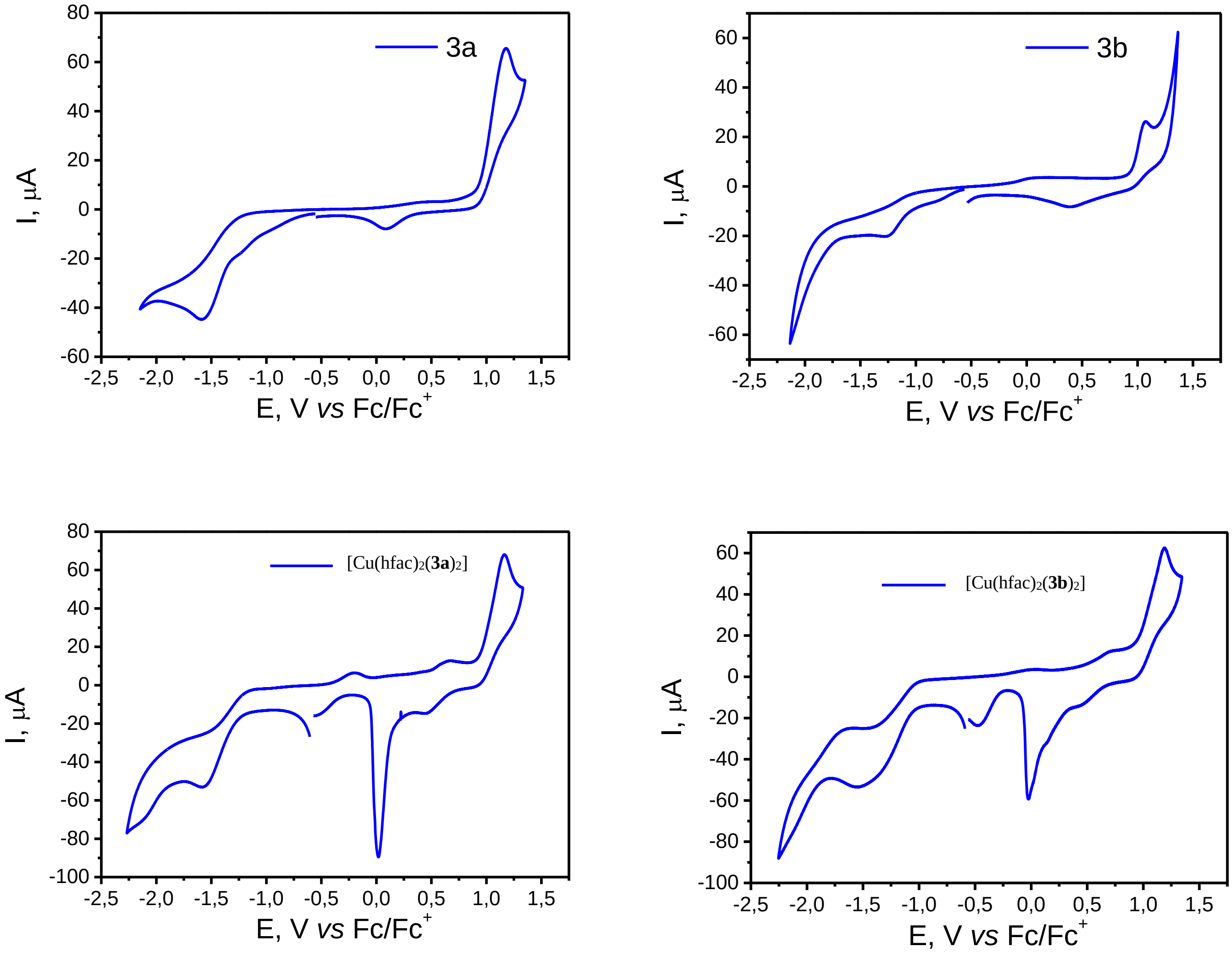

Figure S21. Cyclic voltammograms of nitroxides **3a,b** and complexes [Cu(hfac)$_2$(**3a**)$_2$], [Cu(hfac)$_2$(**3b**)$_2$] in CH_2Cl_2 solution.

Crystallographic data for amine 2b and complex [Cu(hfac)$_2$(2b)$_2$]

Crystallographic data for [Cu(hfac)$_2$(**2b**)$_2$]: $C_{32}H_{22}CuF_{20}N_4O_4$, *M* 970.08, triclinic, P-1, *a* 9.7317(4), *b* 10.5505(4), *c* 10.6014(4) Å; *α* 95.728(2), *β* 101.163(2), *γ* 111.330(2)º; *V* 977.34(7) Å^3, *Z* 1, D_{calcd} 1.648 g·cm^{-3}, *μ*(Mo-*K*α) 0.696 mm^{-1}, F(000) 483, (θ 2.11–30.09°), completeness (θ 50º) 99.9%, *T* = 296(2) K, green, (0.73 × 0.60 × 0.10) mm^3, transmission 0.5079–0.6042, 22970 measured reflections in index range –13 ≤ h ≤13, –14 ≤ k ≤14, –14 ≤ l ≤14, 5708 independent (R_{int} 0.041), 277 parameters, R_1 0.0586 (for 4614 observed $I > 2\sigma(I)$), wR_2 0.1978 (all data), GOOF 1.09, largest diff. peak and hole 0.850 and –0.502 e·A^{-3}

Crystallographic data for **2b**: $C_{11}H_{10}N_2F_4$, *M* 246.21, monoclinic C2/c, *a* 22.849(1), *b* 7.8244(4), *c* 14.4219(6) Å; *β* 120.058(1)º; *V* 2231.6(2) Å^3, *Z* 8, D_{calcd} 1.466 g·cm^{-3}, *μ*(Mo-*K*α) 0.135 mm^{-1}, F(000) 1008, (θ 2.8–30.2°, completeness (θ 50º) 98.2%), *T* = 200(2) K, red, (1.0 × 0.7 × 0.2) mm^3, transmission 0.8129–0.8622, 11034 measured reflections in index range –30 ≤ h ≤ 29, –11 ≤ k ≤ 10, –16 ≤ l ≤ 19, 2780 independent (R_{int} 0.0308), 160 parameters, R_1 0.0465 (for 2415 observed $I > 2\sigma(I)$), wR_2 0.1219 (all data), GOOF 1.05, largest diff. peak and hole 0.336 and –0.210 e·A^{-3}.

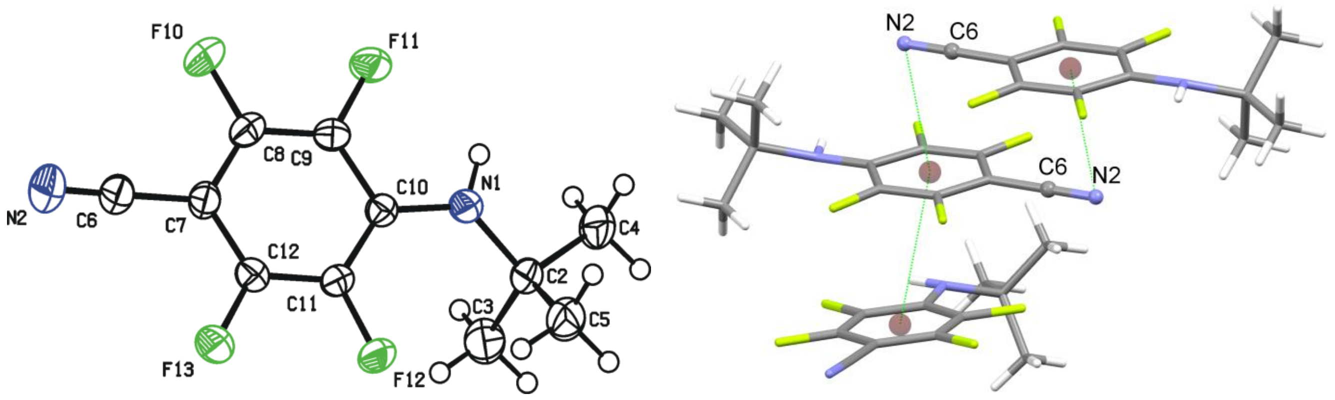

Figure S22. The molecular structure and atom-labelling (left), the fragment of crystal packing of compound **2b** (right) (displacement ellipsoids are drawn at the 50% probability level).

Analysis of the crystal packing of amine **2b** revealed molecular chains along the axis *c* (Figure S21) formed by C≡N…*π* interactions with N…C_g and D_{pln} distances equaling to 3.508(2) and 3.411 Å respectively, and also $\pi^F \ldots \pi^F$ interactions with $C_g \ldots C_g$ and D_{pln} distances being equal to 3.5321(9) and 3.3863(7) Å accordingly. The chains are combined into layers parallel with plane (*b*, *c*) due to weak hydrogen bonds N1-H1N...F13 with H…F, N…F and N–H…F parameters equaling to 2.50(2), 3.354(2) Å and 163(2)º correspondingly.
